# Patient-Centered Digital Health Records and Their Effects on Health Outcomes: Systematic Review

**DOI:** 10.2196/43086

**Published:** 2022-12-22

**Authors:** Martijn R Brands, Samantha C Gouw, Molly Beestrum, Robert M Cronin, Karin Fijnvandraat, Sherif M Badawy

**Affiliations:** 1 Department of Pediatric Hematology, Emma Children’s Hospital Amsterdam Reproduction & Development, Public Health Amsterdam UMC location University of Amsterdam Amsterdam Netherlands; 2 Galter Health Sciences Library at Northwestern University Chicago, IL United States; 3 Department of Medicine The Ohio State University Columbus, OH United States; 4 Department of Molecular Cellular Hemostasis Sanquin Research and Landsteiner Laboratory Amsterdam Netherlands; 5 Department of Pediatrics Feinberg School of Medicine, Northwestern University Chicago, IL United States; 6 Division of Hematology, Oncology, and Stem Cell Transplant Ann and Robert H Lurie Children's Hospital of Chicago Chicago, IL United States

**Keywords:** telemedicine, health records, personal, electronic health records, outcome assessment, health care

## Abstract

**Background:**

eHealth tools such as patient portals and personal health records, also known as patient-centered digital health records, can engage and empower individuals with chronic health conditions. Patients who are highly engaged in their care have improved disease knowledge, self-management skills, and clinical outcomes.

**Objective:**

We aimed to systematically review the effects of patient-centered digital health records on clinical and patient-reported outcomes, health care utilization, and satisfaction among patients with chronic conditions and to assess the feasibility and acceptability of their use.

**Methods:**

We searched MEDLINE, Cochrane, CINAHL, Embase, and PsycINFO databases between January 2000 and December 2021. PRISMA (Preferred Reporting Items for Systematic Reviews and Meta-Analyses) guidelines were followed. Eligible studies were those evaluating digital health records intended for nonhospitalized adult or pediatric patients with a chronic condition. Patients with a high disease burden were a subgroup of interest. Primary outcomes included clinical and patient-reported health outcomes and health care utilization. Secondary outcomes included satisfaction, feasibility, and acceptability. Joanna Briggs Institute critical appraisal tools were used for quality assessment. Two reviewers screened titles, abstracts, and full texts. Associations between health record use and outcomes were categorized as *beneficial*, *neutral or clinically nonrelevant*, or *undesired*.

**Results:**

Of the 7716 unique publications examined, 81 (1%) met the eligibility criteria, with a total of 1,639,556 participants across all studies. The most commonly studied diseases included diabetes mellitus (37/81, 46%), cardiopulmonary conditions (21/81, 26%), and hematology-oncology conditions (14/81, 17%). One-third (24/81, 30%) of the studies were randomized controlled trials. Of the 81 studies that met the eligibility criteria, 16 (20%) were of high methodological quality. Reported outcomes varied across studies. The benefits of patient-centered digital health records were most frequently reported in the category health care utilization on the “use of recommended care services” (10/13, 77%), on the patient-reported outcomes “disease knowledge” (7/10, 70%), “patient engagement” (13/28, 56%), “treatment adherence” (10/18, 56%), and “self-management and self-efficacy” (10/19, 53%), and on the clinical outcome “laboratory parameters,” including HbA_1c_ and low-density lipoprotein (LDL; 16/33, 48%). Beneficial effects on “health-related quality of life” were seen in only 27% (4/15) of studies. Patient satisfaction (28/30, 93%), feasibility (15/19, 97%), and acceptability (23/26, 88%) were positively evaluated. More beneficial effects were reported for digital health records that predominantly focus on active features. Beneficial effects were less frequently observed among patients with a high disease burden and among high-quality studies. No unfavorable effects were observed.

**Conclusions:**

The use of patient-centered digital health records in nonhospitalized individuals with chronic health conditions is potentially associated with considerable beneficial effects on health care utilization, treatment adherence, and self-management or self-efficacy. However, for firm conclusions, more studies of high methodological quality are required.

**Trial Registration:**

PROSPERO (International Prospective Register of Systematic Reviews) CRD42020213285; https://www.crd.york.ac.uk/prospero/display_record.php?RecordID=213285

## Introduction

### Background

The prevalence and disease burden of chronic health conditions is on the rise. The World Health Organization predicts that by 2030, chronic noncommunicable health conditions will account for >50% of the total disease burden [1,2]. In particular, cardiovascular conditions, cancer, respiratory conditions, and diabetes have the highest morbidity and mortality [1]. Currently, 60% of the US population has at least 1 chronic condition and 42% of the population has multiple chronic conditions [3]. This results in a high individual disease burden owing to the large impact on social participation and required patient self-management skills. Self-management refers to a person’s ability to manage the clinical, psychosocial, and societal aspects of their illness and its care [4]. In contrast, self-efficacy is a person’s belief that he or she can successfully execute this behavior [4]. Apart from a high individual disease burden, the prevalence of chronic conditions imposes a high macroeconomic burden [5]. Furthermore, an increasing shortage of health care providers is expected, among others in the United States [6] and Europe [7,8]. In combination with the increased pressure put on health systems by unexpected events such as the COVID-19 pandemic, this shortage threatens the delivery of essential health services [9]. To preserve the access to care for all patients, new technologies are increasingly being developed and adopted, including patient-centered digital health records.

Such patient-centered digital health records can significantly help engage and empower patients with a chronic health condition [10-13]. Patient-centered digital health records enable patients to take on a more active role in their care by allowing them to view parts of their medical records, such as medication lists, laboratory and imaging results, allergies, and correspondence. Other common features include secure messaging, requesting prescription refills, video consultation, paying bills, and managing appointments. Examples of patient-centered digital health records include patient portals and personal health records (PHRs). Patient-centered digital health records differ in the volume and detail of the provided medical data, functionalities, and level of patient control, as shown in Textbox 1. Highly engaged patients are reported to have increased disease knowledge, better self-management, more self-efficacy, and improved clinical outcomes [14-16]. The effects of using patient-centered digital health records may be most substantial for patients with chronic conditions. Many self-management skills are required, and their potential gains are the highest. Not only patients but the entire health care system might benefit from an increased adoption of patient-centered digital health records.

**Proposed taxonomy of patient-centered digital health records [10,17-21].**

- Electronic health record (EHR): a digital version of a health care provider’s paper chart, used by health care professionals alone. Patients cannot access data in an EHR. An EHR might contain data from one health care institution or from multiple institutions. Its scope can range from regional, to national, or international.
- Patient portal: the patient-facing interface of an EHR that enables people to view sections of their medical record. This might include access to test results, medication lists, or therapeutic instructions. Health care providers or health care offices determine what health information is accessible for patients. Patient portals often have additional features such as patient-professional messaging, requesting prescription refills, scheduling appointments, or communicating patient-reported outcomes. By definition, patient portals are “tethered,” in which “tethered” refers to a patient portal’s connection to an EHR. Occasionally, a patient portal is referred to as a tethered personal health record (PHR).
- PHR: a PHR is similar to a patient portal and can have similar features. However, the main difference is that contents are managed and maintained by individuals, not health care providers. People can access, manage, and share their health information, and that of others for whom they are authorized, such as parents or caretakers. Health information from different health care institutions may reside in a single patient-managed PHR. In general, PHRs are not tethered unless otherwise specified. Few tethered PHRs currently exist but are increasingly being developed [22].
- Patient-centered digital health records: an umbrella term referring to patient portals, tethered PHRs, and part of the untethered PHRs. Patient-centered digital health records enable a 2-way exchange of health information between patients and the health care system and provide patients with the ability to view, download, or transmit their health information on the web. This health information is updated at regular intervals. In addition, it enables communication between patients and the health care system, either by adding or editing health information, exchanging patient-reported outcomes, or by using communication tools such as messaging. Additional functionalities are often present.
- “Electronic medical record” is an outdated term [21]. It can be considered a professional-centered EHR with limited functionalities.

Currently, huge investments of time and resources are made in patient-centered digital health records. However, limited insight exists in how the use of patient-centered digital health records by patients with a broad range of chronic conditions affects clinical and patient-reported outcomes and health care utilization. Moreover, we lack an overview of their effects on patient satisfaction, and the feasibility and acceptability of their use by people with chronic conditions. Previous systematic reviews focused on one health condition [23], focused on one type of digital health record [24-27], investigated a select set of health outcomes [24,26,28], or are now obsolete in this rapidly changing technological landscape [23,25,27].

### Objectives

Therefore, in this systematic review, we summarized the available evidence on patient-centered digital health records. Our primary objective was to assess how patient-centered digital health records for nonhospitalized patients with chronic conditions affect clinical and patient-reported health outcomes and health care utilization. Our secondary objective was to evaluate patient satisfaction with and feasibility and acceptability of using patient-centered digital health records. Results of this systematic review may help guide future development and implementation.

## Methods

The protocol for this study was registered in the International PROSPERO (International Prospective Register of Systematic Reviews) Register of Systematic Reviews (CRD42020213285) [29]. The PRISMA (Preferred Reporting Items for Systematic Reviews and Meta-Analyses) guidelines were followed [30].

### Literature Search

A medical librarian (MB) conducted the original literature search using the following databases: MEDLINE, Cochrane Library, CINAHL, Embase, and PsycINFO. All original studies published between January 1, 2000, and December 1, 2020, were assessed. A search update in MEDLINE was performed for all studies published between December 1, 2020, and December 31, 2021. Multimedia Appendix 1 presents the full search strategy. Articles published before 2000 were excluded because of the rapidly changing field of digital health technology [30].

### Eligibility Criteria

Patient-centered digital health records were defined as mobile health (mHealth) or eHealth technologies that enable a 2-way exchange of health information between patients and the health care system, such as patient portals, PHRs, or mHealth apps with a health record functionality. A patient-centered digital health record provides patients with the ability to view, download, or transmit their health information on the web. This health information was updated at regular intervals. In addition, a patient-centered digital health record allows for communication between patients and the health care system, either by adding or editing health information, exchanging patient-reported outcomes, or by using communication tools such as messaging. Several other functionalities are common, but were not considered essential; for example, appointment scheduling, requesting prescription refill, viewing educational material, using decision support tools, and using connected wearables. Exclusion criteria were nondigital health records, digital health records intended for hospitalized patients, and digital health records that are not accessible to patients, such as the clinician-facing components of the electronic health record (EHR).

### Studies

Studies investigating patient-centered digital health records intended for nonhospitalized patients with a chronic health condition were included. Only studies published in English were included. Eligible studies included randomized controlled trials (RCTs), quasi-experimental studies, nonexperimental observational studies (including cohort and cross-sectional studies), and pilot or feasibility studies. Of mixed methods studies, only nonqualitative parts were used for data extraction. Studies that only described health care providers’ experiences were excluded.

### Participants

Studies on patients with a chronic health condition of all age groups were considered. Chronic conditions included all diseases with a moderate to high disease burden and moderate to high impact on daily life. Consequently, these conditions demand considerable self-management skills from patients to manage the clinical, psychosocial, and societal aspects of chronic condition and its care. The selection of chronic conditions included in our search strategy was based on the Charlson Comorbidity Index, other literature, and clinical expertise [31,32]. Diseases included cancer, arthritis, HIV, AIDS, asthma, chronic obstructive pulmonary disease, chronic heart conditions, hematologic disease, chronic kidney disease, celiac disease, inflammatory bowel disease, cystic fibrosis, diabetes mellitus, and multiple sclerosis (MS).

### Outcomes

Studies were required to report at least one primary or secondary outcome. Primary outcomes were clinical outcomes (including disease events and complications, vital parameters, and laboratory parameters), patient-reported outcomes (including self-management and self-efficacy, patient engagement, health-related quality of life (HRQoL), stress and anxiety, and treatment adherence), and health care utilization (including the number of emergency department [ED] visits and hospitalizations, the use of preventive or recommended care services by patients, and regular workload for health care professionals). Secondary outcomes included technology-related outcomes (including patient satisfaction, feasibility, and acceptability). Definitions and examples of these 13 outcomes are presented in Table 1.

**Table 1:** Definitions and examples of all health outcomes included in this systematic review.

Included study outcomes		Definitions and examples
**Clinical outcomes**		
	Disease events and complications	For example, asthma exacerbation, chronic kidney disease progression, and death
	Vital parameters	For example, blood pressure, BMI, weight, and respiratory parameters
	Laboratory parameters	For example, HbA_1c_^a^, LDL^b^, cholesterol, eGFR^c^, HIV viral load, and CD4+ T-cell count
**Patient-reported outcomes**		
	Self-management and self-efficacy	Self-management is a person’s ability to manage the clinical, psychosocial, and societal aspects of illness and its care. Self-efficacy is the belief that a person can successfully execute this behavior (eg, measured by the validated Diabetes Empowerment Scale) [4]
	Patient engagement	Patient engagement comprises 3 suboutcomes: Patient activation: patients believe that their own role in managing their care is important, patients’ confidence and knowledge to take action, how much they take action, and if patients are capable of staying on course under stress (eg, measured by the Patient Activation Measure PAM13) [33] Patient involvement: patients’ involvement and participation in treatment decisions, and patients’ involvement in sharing information, preparing and conducting a medical consultation, and accepting instructions from doctors and nurses [34] (eg, measured by the number of patients that is in possession of an Asthma Action Plan) Disease knowledge: patients’ knowledge of a disease and its related care activities (eg, measured by the Brief Diabetes Knowledge Test) [35]
	Health-related quality of life	All aspects of one’s quality of life that are health-related, including physical functioning, social functioning, and mental health (eg, measured by the 36-Item Short Form Survey SF-36) [36] A reduction in anxiety or stress was considered a suboutcome (eg, measured by the parenting stress index) [37]
	Treatment adherence	The extent to which a person’s behavior (taking medication, following a diet, or the execution of lifestyle changes) corresponds with health care providers’ recommendations [38] (eg, adherence to HIV medication)
**Health care utilization: >all types of encounters between patients and health care providers, including ED^d^ visits, hospitalizations, outpatient clinic appointments, and telephone calls**		
	ED visits and hospitalizations	Reductions in undesirable events (eg, reductions in emergency department visits and hospitalizations)
	Recommended care services	Increased use of recommended care services by people with uncontrolled disease, and the improved use of preventive care services (eg, follow-up outpatient clinic visits among people with uncontrolled HIV, eye examinations in people with diabetes)
	Regular workload	A decrease in regular workload for health care professionals (eg, patients use email instead of interruptive telephone calls as a first method of contact)
**Technology-related outcomes**		
	Patient satisfaction	Patient satisfaction with accessing and using patient-centered digital health records Patient satisfaction with the effects of using patient-centered digital health records (eg, sense of control, perceived quality of care)
	Feasibility	Adherence to patient-centered digital health records and user retention rates, for which no universal cut-off values are available
	Acceptability	The perceived usability of patient-centered digital health records and how these affect behavior, as well as identified facilitators and barriers

^a^HbA_1c_: glycated hemoglobin.

^b^LDL: low-density lipoprotein.

^c^eGFR: estimated glomerular filtration rate.

^d^ED: emergency department.

### Data Extraction

Two independent reviewers (MB and SB) assessed titles, abstracts, and full texts for eligibility. Disagreements were resolved by discussion, if necessary, with a third reviewer (SG).

A modified, electronic version of the standardized Cochrane data extraction form [39] was used to extract the following data items: first author’s name; publication year; study design; disease or diseases studied; study aim; country and setting; participants’ age and sex; sample size; inclusion and exclusion criteria; follow-up duration; description, features, and purpose of the patient-centered digital health record and (if applicable) of the comparator; size and description of the control group (if applicable); device used; description of health outcomes and results; and main study findings.

### Quality Appraisal

For quality appraisal, Joanna Briggs Institute (JBI) critical appraisal tools for RCTs, cross-sectional studies, cohort studies, and quasi-experimental studies were used [40]. JBI tools were modified to better suit the assessment of digital health record studies. Several items were added, including adequate patient-centered digital health record descriptions and selection bias measures, as presented in Multimedia Appendix 2. As the JBI tools differed in the number of items, all scores were converted to a 15-point scale. Articles with a score of ³12 were considered of “high quality,” between 8.5 and 11.9 of “medium quality,” and <8.5 of “low quality.”

### Data Synthesis

Associations between patient-centered digital health record use and health outcomes were categorized in 3 groups: “beneficial,” “neutral or clinically nonrelevant,” or “undesired.” Categorizations were determined by our interpretation of study findings, based on meaningful clinical effects and statistical significance (*P*<.05), and could therefore differ from the authors’ conclusions. Statistical significance was considered relevant only if the effect size were clinically significant. If available, minimal clinically important differences were used to assess effect sizes. The summarization of effects was based on the vote-counting method, as no meta-analysis could be performed. The findings were summarized for all conditions, grouped by disease category (diabetes mellitus, cardiopulmonary diseases, hematology-oncology diseases, and other diseases), and grouped according to outcome type (clinical outcomes, patient-reported outcomes, health care utilization, and technology-related outcomes).

### Subgroup Analyses

Several subgroup analyses were performed. The first subgroup included conditions with a high disease burden. These included conditions with either impaired social participation or that require a high level of self-management skills. Impaired social participation was defined as being unable to participate in work or school or engage with friends and family as desired because of the condition or its treatment. High self-management skills are defined as recurrent actions demanded from patients to prevent or treat the disease or its consequences, including high disease-related knowledge needed to actively engage in decision-making. This subgroup was determined based on clinical expertise of the study team. Second, we assessed 2 subgroups: patient-centered digital health records that predominantly offered passive features and those that predominantly offered active features. Passive features are those through which the patient receives information but does not actively add information. Active features are those in which the patient performs an action and actively engages with the digital health record. The third subgroup of interest included studies with high methodological quality. A sensitivity analysis was performed to investigate whether our results were influenced by poor quality studies. Finally, the subgroups of interest were studies that included older participants (mean age >55 years), a high number of female participants (>45%), or a racially diverse population (<50% White participants).

## Results

### Overview

**Figure 1:**
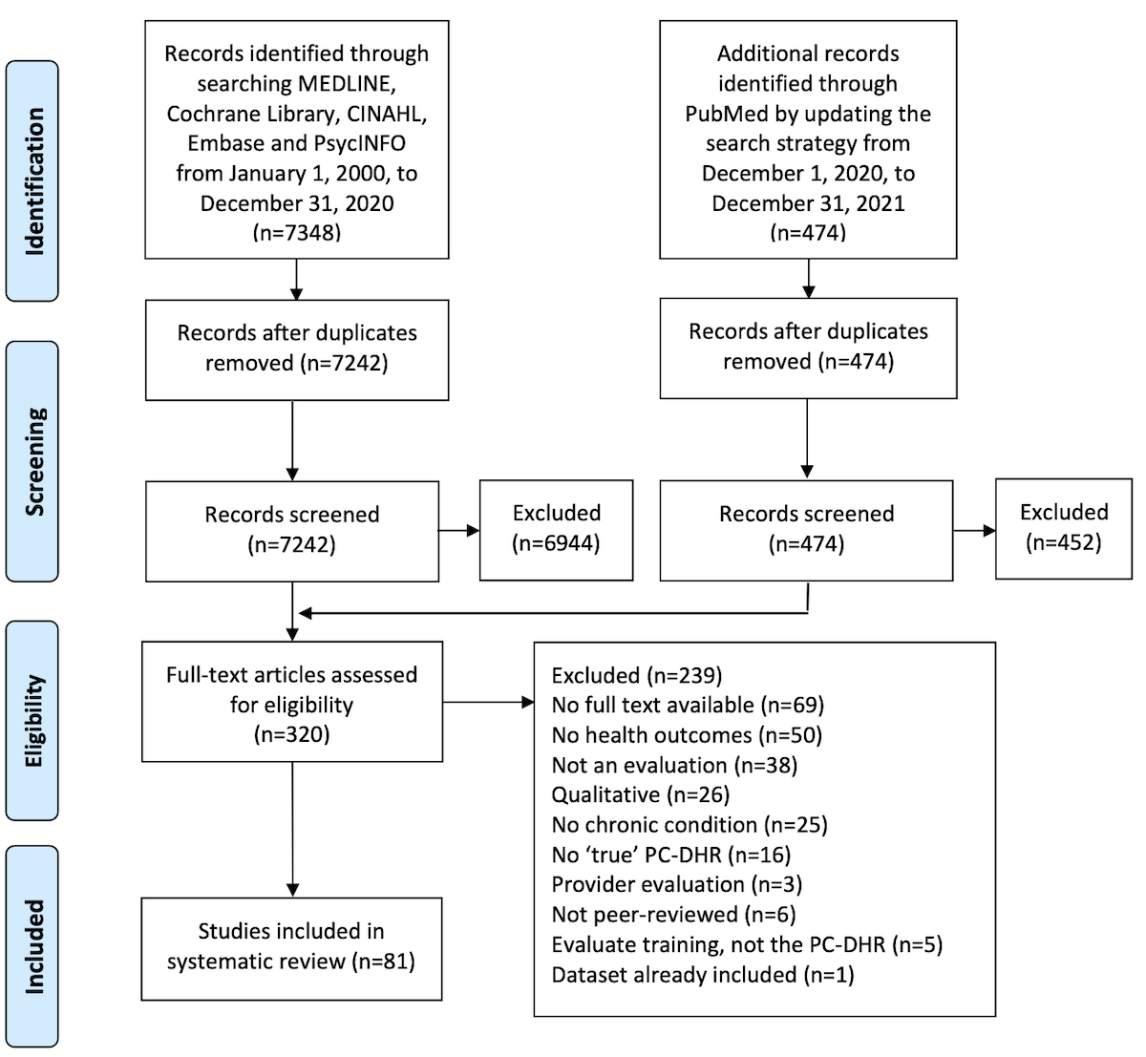
PRISMA (Preferred Reporting Items for Systematic Reviews and Meta-Analyses) flow diagram. PC-DHR: patient-centered digital health record.

The search yielded 7716 unique publications. After screening the titles and abstracts, 320 full-text articles were retrieved. A total of 81 articles met the inclusion criteria. No non-English articles that met the inclusion criteria were identified. Figure 1 shows the study PRISMA flowchart. In total, 1,639,556 participants were included in the studies of this systematic review. Most (74/81, 91%) studies included only adult participants. Of the total 1,369,913 participants, 99% (n=1,629,660) were adults. Nine studies included children or their parents, with a total number of 9297 children and 599 parents. Sample sizes of studies varied from 10 to 267,208 participants. Furthermore, 46% (747,370/1,639,556) of the participants were female. Of the 81 included studies, health literacy was reported by 7 (9%) studies and insurance status by 15 (20%) studies. Race distribution was reported by 74% (60/81) of studies, of which 47 (78%) studies included a population of which more than half were White and 26 (43%) studies of which >75% were White.

### Study Characteristics

Study characteristics are presented in Tables 2-5 (36 studies are listed in Table 2; 11 studies are listed in Table 3, 14 studies are listed in Table 4, and 20 studies are listed in Table 5). Most investigated conditions were type 1 or 2 diabetes mellitus (37/81, 46%), cardiovascular conditions (14/81, 17%), and malignancies (11/81, 14%). Studies were mostly conducted in the following countries: United States (58/81, 72%), the Netherlands (7/81, 9%), Canada (5/81, 6%), and United Kingdom (3/81, 4%). In addition, 30% (24/81) of the studies were RCTs, 27% (22/81) were cross-sectional studies, 20% (16/81) were retrospective observational cohort studies, and 23% (18/81) were quasi-experimental studies, including pretest-posttest and feasibility studies. One study was a secondary data analysis of the intervention group in an RCT. Of the 55 studies that reported follow-up durations, 6 (7%) studies had a follow-up of less than a month, 25 (31%) studies between 1 and 6 months, 14 (17%) studied between 7 and 12 months, and 10 (12%) studies of >12 months.

Explanations of the patient-centered digital health records investigated in each study are presented in Tables 6-9. Patient-centered digital health records range from a pilot patient portal enabling patients to view a limited set of their medical data to comprehensive PHRs, offering extensive data access and enabling appointment scheduling and prescription refill requests. A minority (12/81, 15%) of studies specifically evaluated ≥1 digital health record features such as secure messaging or a medication adherence module. In addition, 15% (12/81) of studies used a hybrid approach to assess a combination of a digital health record with a connected device, or with training, coaching, or face-to-face visits.

**Table 2:** Study characteristics of studies investigating diabetes mellitus (of 37 studies investigating diabetes mellitus, 36 are listed in Table 2).^a^

Author, year	Country, setting	Study population, disease, controlled?	Burden^b^	Study design	Sample size	Age (years)^c^, mean (SD)	Gender^c^ (female), n (%)	Race^c^ (White), n (%)
Bailey et al [41], 2019	United States, 2 academic hospitals	Adults with DM^d^, on high-risk medication	−	Pilot or feasibility	100	56 (11)	57 (57)	48 (48)
Boogerd et al [42], 2017	Netherlands, 7 medical centers	Parents of children <13 years with DM type 1	+	Pilot or feasibility	I^e^=54, C^f^=51	9.1 (2.7): Children	30 (56)	NR^g^
Byczkowski et al [43], 2014	United States, 1 academic hospital	Parents of children with DM (or CF^h^ or JIA^i^)	±	Cross-sectional	I=126, C=89	11 (NR)	69 (54.8)	115 (91.3)
Chung et al [44], 2017	United States, outpatient care organization	Adults with DM	−	Cohort	I=12,485, C=2831	56 (12)	5493 (44)	5119 (41)
Conway et al [45], 2019	United Kingdom, Scotland’s health system	Patients with DM	−	Cross-sectional	1095	58 (12)	405 (36.99)	873 (78.73)
Devkota et al [46], 2016	United States, 6 PCPs^j^	Patients with DM type 2	−	Cohort	I=409, C=1101	58 (12)^k^	235 (57.5)	250 (61.1)
Dixon et al [47], 2016	United States, 3 community centers	Adults with DM type 2	−	Pilot or feasibility	96	53 (11)	56 (58)	47 (49)
Graetz et al [48], 2018	United States, integrated health system	Adults with DM	−	Cross-sectional	267,208	NR	127,458 (47.7)	116,770 (43.7)
Graetz et al [49], 2020	United States, integrated health system	Adults with DM with at least 1 oral drug	−	Cross-sectional	111,463	64 (13)	51,545 (46.24)	45,205 (40.56)
Grant et al [50], 2008	United States, 11 PCPs	Adults with DM using medication	−	RCT^l^	I=126, C=118	59 (10)	54 (42.9)	117 (92.9)
Lau et al [51], 2014	Canada, 1 academic hospital	Adults with DM	−	Cohort	I=50, C=107	55 (14)	22 (44)	NR
Lyles et al [52], 2016	United States, integrated health system	Adults with DM type 2 using statins	−	Cohort	I=8705, C=9055	61 (11)^k^	4013 (46.1)	3134 (36)^k^
Martinez et al [53], 2021	United States, 4 medical centers	Adults with DM type 2 using medication	−	Pilot or feasibility	60	58 (13)	33 (55)	41 (68)
McCarrier et al [54], 2009	United States, 1 diabetes clinic	Adults <50 years with uncontrolled DM type 1	+	RCT	I=41, C=36	57 (8)	15 (37)	39 (95)
Osborn et al [55], 2013	United States, 1 academic hospital	Adults with DM type 2 using medication	−	Cross-sectional	I=62, C=13	57 (8)	39 (63)	46 (74)
Price-Haywood and Luo [56], 2017	United States, integrated health system	Adults with DM or HT^m^	−	Cohort	I=10,497, C=90,522	NR	6205 (59.11)	8055 (76.74)
Price-Haywood et al [57], 2018	United States, integrated health system	Adults with DM or HT	−	Cohort	I=11,138, C=89,880	58 (13)	6,204 (55.7)	NR
Quinn et al [58], 2018	United States, 26 PCPs	Adults <65 years with DM type 2	−	RCT	I=82, C=25	54 (8)	39 (48)	51 (62)
Reed et al [59], 2015	United States, integrated health system	Adults with DM, HT, CAD^n^, asthma, or CHF^o^	±	Cross-sectional	1041	NR	587 (56.4)	618 (59.4)
Reed et al [60], 2019	United States, integrated health system	Adults with DM+HT, CAD, asthma, or CHF	±	Cross-sectional	165,477	NR	79,594 (48.1)	NR (60.9)
Reed et al [61], 2019	United States, integrated health system	Adults with DM, asthma, HT, CAD, CHF or CV event risk	±	Cross-sectional	I=1392, C=407	NR	719 (51.7)	816 (58.6)
Riippa et al [62], 2014	Finland, 10 PCPs	Adults with DM, HT or HC^p^	−	RCT	I=80, C=57	61 (9)	45 (56)	NR
Riippa et al [63], 2015	Finland, 10 PCPs	Adults with DM, HT or HC	−	RCT	I=80, C=57	61 (9)	45 (56)	NR
Robinson et al [64], 2020	United States, 1 veteran hospital	Veterans with uncontrolled DM type 2	−	Cross-sectional	I=446, C=754	66 (8)	28 (6.3)	384 (86.1)
Ronda et al [65], 2014	Netherlands, 62 PCPs+1 hospital	Adults with DM	−	Cross-sectional	I=413, C=758	64 (12)	154 (37.3)	383 (93.6)
Ronda et al [66], 2015	Netherlands, 62 PCPs+1 hospital	Adults with DM	−	Cross-sectional	I=413, C=219	59 (13)	154 (37.3)	383 (93.6)
Sabo et al [67], 2021	United States, 21 practices	Adults with DM type 2	−	Cohort	I=189, C=148	61 (13)	75 (40.9)	113 (72.9)
Sarkar et al [68], 2014	United States, integrated health system	Adults with DM	−	Cohort	I=8705, C=9055	61 (11)^k^	4013 (46.1)	5072 (58.27)
Seo et al [69], 2020	South Korea, 1 academic hospital	Patients with DM	−	Cohort	I=133, C=7320	54 (10)	23 (17.3)	NR
Sharit et al [70], 2018	United States, 1 veterans center	Overweight veterans with prediabetes	−	Pilot or feasibility	38	58 (8)	9 (24)	8 (21)^k^
Shimada et al [71], 2016	United States, Veteran registry	Veterans with uncontrolled DM, HT or LDL^q^	−	Cohort	I=50,482, C=61,204	61 (10)	2060 (4.08)	35,761 (70.84)
Tenforde et al [72], 2012	United States, 1 community hospital	Adults <75 years with DM	−	Cohort	I=4036, C=6710	59 (10)	1857 (46)^k^	3,390 (84)^k^
van Vugt et al [73], 2016	Netherlands, 52 PCPs	Patients with DM type 2	−	RCT	I=66, C=66	68 (10)	54 (41)	91 (69)
Vo et al [74], 2019	United States, integrated health system	Adults <80 years with DM type 2	−	RCT	I=673, C=603	61 (10)	296 (44)	394 (58.5)
Wald et al [75], 2009	United States, 230 PCPs	Patients with DM type 2	−	RCT	126	59 (NR)	53 (42.1)	117 (92.9)
Zocchi et al [76], 2021	United States, nationwide	Patients with DM type 2, partly uncontrolled	−	Cohort	95,043	63 (10)	4,339 (4.57)	68,954 (72.55)

^a^All studies are listed in Tables 2-5 and are reported in the disease category of the condition that is most prominently investigated. The study by Druss et al [77] is therefore listed in Table 5.

^b^If conditions are considered to have a high disease burden or demand high self-management skills, a positive sign is shown. Otherwise, a sign is indicated. A ± sign indicates that multiple diseases have been studied, and only some of the diseases were considered to have a high disease burden.

^c^If available, age (years), gender, and race were reported by digital health record users (“the intervention group”).

^d^DM: diabetes mellitus.

^e^I: intervention.

^f^C: control.

^g^NR: not reported.

^h^CF: cystic fibrosis.

^i^JIA: juvenile idiopathic arthritis.

^j^PCP: primary care practice.

^k^Presented numbers were estimated based on the data provided in the original articles.

^l^RCT: randomized controlled trial.

^m^HT: hypertension.

^n^CAD: coronary artery disease.

^o^CHF: congestive heart failure.

^p^HC: hypercholesterolemia.

^q^LDL: low-density lipoprotein.

**Table 3:** Study characteristics of studies investigating cardiopulmonary diseases (of 21 studies investigating cardiopulmonary diseases, 11 are listed in Table 3).^a^

Author, year	Country, setting	Study population, disease, controlled?	Burden^b^	Study design	Sample size	Age (years)^c^, mean (SD)	Gender^c^ (female), n (%)	Race^c^ (White), n (%)
Aberger et al [78], 2014	United States, renal transplant clinic	Postrenal transplant patients with HT^d^	+	Pilot or feasibility	66	54 (NR^e^)	34 (52)^f^	48 (72)^f^
Ahmed et al [79], 2016	Canada, 2 academic hospitals	Adults with asthma using medication	+	RCT^g^	I^h^=49, C^i^=51	NR	32 (68)	NR
Apter et al [80], 2019	United States, multicenter hospitals	Adults with asthma using prednisone	+	RCT	I=151, C=150	49 (13)	270 (89.7)	4 (1.3)
Fiks et al [81], 2015	United States, 3 PCPs^j^	Children aged 6-12 years with asthma, partly uncontrolled	+	RCT	I=30, C=30	8.3 (1.9)	26 (87) among parents	13 (43)
Fiks et al [82], 2016	United States, 20 PCPs	Children aged 6-12 years with asthma, partly uncontrolled	+	Pilot or feasibility	I=237, C=8896	NR	101 (42.8)	144 (61.5)
Kogut et al [83], 2014	United States, 1 community hospital	Adults aged >49 years with cardiopulmonary disorders	±	Pilot or feasibility	30	NR	14 (47)	NR
Kim et al [84], 2019	South Korea, 1 academic hospital	Patients with obstructive sleep apnea	−	RCT	I=30, C=13	43 (10)^f^	NR (15)	NR
Lau et al [85], 2015	Australia, nationwide	Adults with asthma	+	RCT	I=154, C=176	40 (14)	124 (80.5)	NR
Manard et al [86], 2016	United States, PCP registry	Adults with uncontrolled HT	−	Cohort	I=400, C=1171	61 (12)	262 (65.5)	72
Toscos et al [87], 2020	United States, 1 community hospital	Patients with nonvalvular AF^k^ with OAC^l^	+	RCT	I=76, C=77	71 (9)	60 (37.5)	153 (99.4)
Wagner et al [88], 2012	United States, 24 PCPs	Patients with hypertension, partly uncontrolled	−	RCT	I=193, C=250	55 (12)	145 (75.1)	96 (50.5)

^a^All studies are listed in Tables 2-5 and are reported in the disease category of the condition that is most prominently investigated. The studies by Price-Haywood and Luo [56], Price-Haywood et al [57], Reed et al [59], Reed et al [60], Reed et al [61], Riippa et al [62], Riippa et al [63], Shimada et al [71] are listed in Table 2. The study by Martinez Nicolás et al [89] is listed in Table 4. The study by Druss et al [77] is therefore listed in Table 5.

^b^If conditions are considered to have a high disease burden or demand high self-management skills, a positive sign is shown. Otherwise, a sign is indicated. A ± sign indicates that multiple diseases have been studied, and only some of the diseases were considered to have a high disease burden.

^c^If available, age (years), gender, and race were reported by digital health record users (“the intervention group”).

^d^HT: hypertension.

^e^NR: not reported.

^f^Presented numbers were estimated based on the data provided in the original articles.

^g^RCT: randomized controlled trial.

^h^I: intervention.

^i^C: control.

^j^PCP: primary care practice.

^k^AF: atrial fibrillation.

^l^OAC: oral anticoagulant drug.

**Table 4:** Study characteristics of studies investigating hematological and oncological diseases (n=14).

Author, year	Country, setting	Study population, disease, controlled?	Burden^a^	Study design	Sample size	Age (years)^b^, mean (SD)	Gender^b^ (female), n (%)	Race^c^ (White), n (%)
Cahill et al [90], 2014	United States, cancer center	Adults with glioma	+	Cross-sectional	186	44 (13)	87 (46.8)	149 (86.1)
Chiche et al [91], 2012	France, 1 community hospital	Adults with ITP^c^	±	RCT^d^	I^e^=28, C^f^=15	48 (15)^g^	21 (75)	NR^h^
Collins et al [92], 2003	United Kingdom, hemophilia centers	Patients with hemophilia >11 years	+	Pilot or feasibility	10	NR	NR	NR
Coquet et al [93], 2020	United States, cancer center	Patients with cancer+chemotherapy	+	Cohort	I=3223, C=3223	59 (15)	1,554 (49.78)	1,804 (49.68)
Groen et al [94], 2017	Netherlands, cancer center	Patients with lung cancer	+	Pilot or feasibility	37	60 (8)	16 (47)	37 (100)
Hall et al [95],2014	United States, Cancer Center	Patients with resection for CRC^i^ or EC^j^	+	Pilot or feasibility	49	59 (12)^g^	37 (76)	48 (98)
Hong et al [96], 2016	United States, academic pediatric hospital	Children aged 13-17 years with cancer or a blood disorder+parents	+	Cross-sectional	46	15 (1.2)^g^	10 (63) among children	NR
Kidwell et al [97], 2019	United States, multicenter hospitals	Patients aged 13-24 years with sickle cell disease	+	Pilot or feasibility	44	19 (NR)	24 (55)	0 (0)
Martinez Nicolás et al [89], 2019	Spain, 4 community hospitals	Patients with COPD^k^, CHF^l^, or hematologic malignancy	+	Pilot or feasibility	577,121	42 (23)	319,725^g^ (55)	NR
O’Hea et al [98], 2021	United States, cancer centers	Adult women with nonmetastatic breast cancer ending treatment	+	RCT	I=100, C=100	61 (11)	100 (100)	85 (85)
Pai et al [99], 2013	Canada, cancer center	Adult men with prostate cancer	+	Cross-sectional	17	64 (7)^g^	0 (0)	16 (95)
Tarver et al [100], 2019	United States, academic hospital	Patients with colorectal cancer	+	Cross-sectional	22	58 (10)	10 (45)	NR
Wiljer et al [101], 2010	Canada, breast cancer registry	Patients with breast cancer	+	Pilot or feasibility	311	NR	303 (99.7)	NR
Williamson et al [102], 2017	United States, pediatric cancer center	Pediatric cancer survivors	+	Cohort	56	NR	27 (48)	49 (88)

^a^If conditions are considered to have a high disease burden or demand high self-management skills, a positive sign is shown. Otherwise, a sign is indicated. A ± sign indicates that multiple diseases have been studied, and only some of the diseases were considered to have a high disease burden.

^b^If available, age (years), gender, and race were reported by digital health record users (“the intervention group”).

^c^ITP: idiopathic thrombocytopenic purpura.

^d^RCT: randomized controlled trial.

^e^I: intervention.

^f^C: control.

^g^Presented numbers were estimated based on the data provided in the original articles.

^h^NR: not reported.

^i^CRC: colorectal cancer.

^j^EC: endometrial cancer.

^k^COPD: chronic obstructive pulmonary disease.

^l^CHF: congestive heart failure.

**Table 5:** Study characteristics of studies investigating other diseases (of 21 studies investigating other diseases, 20 are listed in Table 5). Diseases include kidney disease (n=3, 15%), mental health disorders (n=3, 15%), multiple sclerosis (n=2, 10%), inflammatory bowel disease (n=2, 10%), rheumatologic conditions (n=2, 10%), and others (n=8, 40%).^a^

Author, year	Country, setting	Study population, disease, controlled?	Burden^b^	Study design	Sample size	Age (years)^c^, mean (SD)	Gender^c^ (female), n (%)	Race^c^ (White), n (%)
Anand et al [103], 2017	Thailand, HIV clinic	MSM^d^ and transgender women with HIV, partly uncontrolled	+	RCT^e^	186	30 (10)^f^	7 (4)	0 (0)
Bidmead and Marshall [104], 2016	United Kingdom, 1 community hospital	Patients with IBD^g^	+	Cross-sectional	60	NR^h^	NR	NR
Crouch et al [105], 2015	United States, 1 HIV clinic	Veterans with HIV, partly uncontrolled	+	Cross-sectional	I^i^=20, C^j^=20	43 (11)	1 (5)	19 (95)
Druss et al [106], 2014	United States, 1 mental health center	Patients with a mental disorder+chronic condition	+	RCT	I=85, C=85	49 (7)	42 (49)	13 (15)
Druss et al [77], 2020	United States, 2 mental health centers	Patients with a mental disorder+DM^k^, HT^l^, or HC^m^	+	RCT	I=156, C=155	51 (6.5)	95 (61)	29 (19)
Jhamb et al [107], 2015	United States, 4 nephrology clinics	Adults visiting nephrology clinics, partly uncontrolled	+	Cross-sectional	1098	58 (16)	549 (50)	952 (86.7)
Kahn et al [108], 2010	United States, HIV clinic	Patients with HIV or AIDS	+	Pilot or feasibility	136	NR	15 (11)^f^	106 (78)^f^
Keith McInnes et al [109], 2013	United States, 8 Veteran hospitals	Veterans with HIV, partly uncontrolled	+	Cross-sectional	1871	NR	51 (2.73)	342 (18.28)
Keith McInnes et al [110], 2017	United States, Veterans care system	Veterans with HIV+detectable viral load, partly uncontrolled	+	Cohort	3374	NR	128 (3.79)	1130 (33.49)
Kiberd et al [111], 2018	Canada, dialysis clinic	Adult with home dialysis	+	Pilot or feasibility	41	57 (2)	13 (48)	NR
Lee et al [112], 2017	South Korea, 1 surgery department	Patients with cleft lip or cleft palate surgery	−	Pilot or feasibility	50	36 (NR)	33 (66)	NR
Miller et al [113], 2011	United States, MS^n^ clinic	Patients with MS	+	RCT	I=104, C=102	48 (9)	73 (71.6)	80 (78.4)
Navaneethanet al [114], 2017	United States, multiple health centers	Adults with chronic kidney disease, partly uncontrolled	+	RCT	I=152, C=57	68 (NR)^f^	79 (52)	117 (77)
Plimpton [115], 2020	United States, HIV clinic	Women with HIV, partly uncontrolled	+	Pilot or feasibility	22	41 (11)	22 (100)	7 (32)
Reich et al [116], 2019	United States, 1 community hospital	Adults with IBD^o^	+	RCT	I=64, C=63	42 (16)	28 (46)	48 (77)
Scott Nielsen et al [117], 2012	United States, 1 academic center	Adults with MS	+	Cross-sectional	I=120, C=120	45 (11)	90 (75)	115 (95.8)
Son and Nahm [118], 2019	United States, online senior community	Patients >49 years with 1 or more chronic conditions	±	Secondary data analysis	272	70 (9)	191 (70.2)	213 (78.3)
Tom et al [119], 2012	United States, integrated health system	Parents of children age <6 years with 1 or more chronic conditions	±	Cross-sectional	I=166, C=90	3 (1)	66 (39.8)	113 (68.1)
van den Heuvel et al [120], 2018	Netherlands, 3 hospitals	Adults with bipolar disorder	+	Cross-sectional	39	45 (11)	44 (67)	NR
van der Vaart et al [121], 2014	Netherlands, 1 hospital	Patients with rheumatoid arthritis	+	Cross-sectional	214	62 (13)	140 (65.4)	NR

^a^All studies are listed in Tables 2-5 and are reported in the disease category of the condition that is most prominently investigated. The study by Byczkowski et al [43] is therefore listed in Table 2.

^b^If conditions are considered to have a high disease burden or demand high self-management skills, a positive sign is shown. Otherwise, a sign is indicated. A ± sign indicates that multiple diseases have been studied, and only some of the diseases were considered to have a high disease burden.

^c^If available, age (years), gender, and race were reported by digital health record users (“the intervention group”).

^d^MSM: men who have sex with men.

^e^RCT: randomized controlled trial.

^f^Presented numbers were estimated based on the data provided in the original articles.

^g^IBD: inflammatory bowel disease.

^h^NR: not reported.

^i^I: intervention.

^j^C: control.

^k^DM: diabetes mellitus.

^l^HT: hypertension.

^m^HC: hypercholesterolemia.

^n^MS: multiple sclerosis.

^o^IBD: inflammatory bowel disease.

**Table 6:** Patient-centered digital health record descriptions for disease category diabetes mellitus (of 37 studies investigating diabetes mellitus, 36 are listed in Table 6).^a^

Author, year		Name		Type		What is evaluated?^b^		Passive features		Active features		Focus^c^
Bailey et al [41], 2019	Electronic Medication Complete Communication		PP^d^		Adherence module alone		View health information (medical summary), read after-visit summary, read educational material		Report medication concerns, monitor medication use		Active	
Boogerd et al [42], 2017	Sugarspace		PP		PP		View treatment goals, read educational material		Parent-professional communication, peer support		Active	
Byczkowski et al [43], 2014	In-house developed		PP		PP		View health information (including laboratory results, medication), view appointments, read disease-specific information		Messaging, upload documents, receive reminders		Passive	
Chung et al [44], 2017	Not reported		PP		Messaging		View health information		Messaging		Active	
Conway et al [45], 2019	My Diabetes My Way		Tethered PHR^e^		PHR		View health information from primary and secondary care (including clinical parameters, medication, and correspondence), read educational material		Report self-measurements		Passive	
Devkota et al [46], 2016	MyChart		PP		PP		View health information (including laboratory results, diagnoses, medication, vital signs), read educational material		Messaging, request prescription refills, schedule appointments, pay bills		Passive	
Dixon et al [47], 2016	CareWeb		PP		Medication module alone		View health information (including measurements, medication)		Report barriers to medication adherence		Passive	
Graetz et al [48], 2018 and Graetz et al [49], 2020	“Kaiser Permanente portal”		PP		PP		View health information (including laboratory results)		Messaging, schedule appointments, request prescription refills, pay bills		Active	
Grant et al [50], 2008	Not reported		PP		PP		View health information (including medication, laboratory results)		Edit medication lists, messaging, report adherence barriers or adverse effects		Active	
Lau et al [121], 2014	BCDiabetes		PP		PP		View health information (including laboratory results), view care plan, read educational material		Messaging, use a journal		Passive	
Lyles et al [52], 2016	“Kaiser Permanente portal”		PP		Medication module alone		View health information (including medical history, laboratory results, and visit summaries)		Messaging, schedule appointments, request prescription refills		Active	
Martinez et al [53], 2021	My Diabetes Care, part of My Health at Vanderbilt		PP		Diabetes module		View health information (including laboratory results and vaccinations), visualize information, read educational material		Messaging, peer support, decision support tools		Active	
McCarrier et al [54], 2009	Living with Diabetes Intervention		PP		PP+case manager		View health information (including correspondence, action plans, and laboratory results), read diabetes-related information		Upload blood glucose readings, use a journal		Active	
Osborn et al [55], 2013	My Health At Vanderbilt		PP		PP		View health information (including vital signs, laboratory results, and medication), read educational information		Messaging, manage appointments, use health screening tools, pay bills		Passive	
Price-Haywood and Luo [56], 2017 and Price-Haywood et al [57], 2018	MyOchsner		PP		PP		View health information (including an after-visit summary, allergies, and laboratory results)		Messaging, request prescription refills, schedule appointments		Passive	
Quinn et al [58], 2018	Not reported		PP		PP		View self-reported health information (including medication and measurements), read educational material		Messaging, report self-measurements and medication changes, receive automated feedback		Active	
Reed et al [59], 2015	“Kaiser Permanente portal”		PP		Messaging alone		View health information (including laboratory results and correspondence)		Messaging, request prescription refills, schedule appointments		Active	
Reed et al [60], 2019 (1) and Reed et al [61], 2019	“Kaiser Permanente portal”		PP		PP		View health information from primary care and secondary care (including laboratory results and visit summaries)		Messaging, request prescription refills, schedule visits		Passive	
Riippa et al [62], 2014 and Riippa et al [63], 2015	Not reported		PP		PP		View health information (including diagnoses, laboratory results, vaccinations, and medication), view care plan, read educational material		Messaging		Passive	
Robinson et al [64], 2020	My HealtheVet		PP		Messaging alone		View health information (including medication and correspondence), view appointments		Messaging, request prescription refills, receive reminders, upload notes and measurements, use a journal		Passive	
Ronda et al [65], 2014 and Ronda et al [66], 2015	Digitaal logboek		PP		PP		View diabetes-specific health information (including laboratory results, diagnoses, and medication), view treatment goals, view appointments		Messaging, upload self-measurements		Passive	
Sabo et al [67], 2021	Diabetes Engagement and Activation Platform		PP		PP		View health information (including medication and self-reported glucose measurements)		Report diet, physical activity, blood glucose measurements, complications, mental health and goals, receive alerts		Active	
Sarkar et al [68], 2014	“Kaiser Permanente portal”		PP		PP		View health information (including medical history, laboratory results, and visit summaries), view appointments		Messaging, request prescription refills		Passive	
Seo et al [69], 2020	My Chart in My Hand		Tethered PHR		PHR+sugar function		View health information (including laboratory results, medication, allergies, diagnoses)		Edit information, schedule appointment; sugar function: log treatment, food intake, and exercise		Active	
Sharit et al [70], 2018	My HealtheVet		PP		Track Health module+wearable		View health information (including medication and correspondence), view appointments		Messaging, request prescription refills, receive reminders; track Health module: record diet and activity, upload data from connected accelerometer		Active	
Shimada et al [71], 2016	My HealtheVet		PP		Messaging, prescription refills		View health information (including medication and correspondence), view appointments		Messaging, request prescription refills, receive reminders, upload notes and self-measurements, use a journal		Active	
Tenforde et al [72], 2012	MyChart		PP		PP		View health information (including diagnoses and laboratory results), read diabetes educational material		Messaging, view glucometer readings, receive reminders		Passive	
van Vugt et al [73], 2016	e-Vita		Tethered PHR		PHR+personal coach		View health information (measurements), read diabetes education		Messaging, self-management support program for personal goal setting and evaluation		Active	
Vo et al [74], 2019	“Kaiser Permanente portal”		PP		PP+PreVisit Prioritization messaging		View health information (including medical history, laboratory results, and visit summaries), view appointments		PreVisit Prioritization messaging to report priorities before a clinic visit, request prescription refills		Active	
Wald et al [75], 2009	Patient Gateway		Tethered PHR		PHR		View health information (including medication, allergies, and laboratory results)		Suggest corrections, report care concerns, ask for referrals, create care plans before visits		Active	
Zocchi et al [76], 2021	My HealtheVet		PP		PP		View health information (including medication, laboratory results, imaging, and correspondence)		Messaging, requesting prescription refills, download health information		Active	

^a^All studies are listed once in Tables 2-5 and are reported in the disease category of the condition that is most prominently investigated. We have included only the functionalities that the authors have reported in their articles. We have applied the taxonomy as presented in Textbox 1 on the information provided by the authors. Therefore, our classification of patient-centered digital health records might not correspond with the term used by the authors.

^b^In this column, we indicated whether authors evaluated the complete patient-centered digital health record, or only part of it.

^c^By definition, patient-centered digital health records have both passive and active features. In this column, we indicate whether patient-centered digital health records predominantly offer passive or active features. In passive features, patients receive information but do not actively add it. In terms of active features, patients perform an action and actively engage with the portal.

^d^PP: patient portal.

^e^PHR: personal health record.

**Table 7:** Patient-centered digital health record descriptions for disease category cardiopulmonary diseases (of 21 studies investigating cardiopulmonary diseases, 11 are listed in Table 7).^a^

Author, year		Name		Type		What is evaluated?^b^		Passive features		Active features		Focus^c^
Aberger et al [78], 2014	Good Health Gateway		PP^d^		PP+BP^e^ cuff		View BP measurements, view treatment goals		Communicate self-reported adherence, receive automated and tailored feedback		Active	
Ahmed et al [79], 2016	My Asthma Portal		PP		PP		View health information (including medication and diagnoses), read general and tailored asthma information		Monitor and receive feedback on self-management practices		Passive	
Apter et al [80], 2019	MyChart		PP		PP		View health information (including laboratory results, vaccinations, and medication), view appointments		Messaging, request prescription refills, schedule appointments		Passive	
Fiks et al [81], 2015 and Fiks et al [82], 2016	MyAsthma		PP		PP		View care plan, read educational material		Report symptoms, treatment adherence, concerns and side effects		Active	
Kim et al [84], 2019	MyHealthKeeper		Tethered PHR^f^		PHR+activity tracker		View previously uploaded self-reported data		Upload self-reported data (eg, diet, sleep, weight, BP, step count), connect with wearables, receive feedback from health care providers		Active	
Kogut et al [83], 2014	ER-Card		Untethered PHR		PHR+home visits by pharmacists		View patient-reported medication list		Pharmacists view and review patient-reported medication lists, and discuss potential concerns in home visits		Active	
Lau et al [85], 2015	Healthy.me		Untethered PHR		PP+extra feature		View Asthma Action Plan, read educational content		Schedule appointments, peer support, self-report medication, use a journal		Passive	
Manard et al [86], 2016	Not reported		PP		PP+BP cuff		View health information (including laboratory results, vital signs, and diagnoses)		Messaging, request prescription refills, upload measurements from connected BP cuff		Passive	
Toscos et al [87], 2020	MyChart		PP		PP+smart pill bottle		View health information (including laboratory results, vaccinations, and medication), view appointments		Messaging, request prescription refills, schedule appointments Smart Pill Bottle: a device that sends notifications when a user opens or fails to open the lid, based on the dose schedule		Active	
Wagner et al, 2012 [88]	MyHealthLink		Tethered PHR		PHR		View health information (including diagnoses, medication, and allergies), read educational material		Messaging, goal setting, upload self-measurements (including BP)		Active	

^a^All studies are listed once in Tables 2-5 and are reported in the disease category of the condition that is most prominently investigated. We have included only the functionalities that the authors have reported in their articles. We have applied the taxonomy as presented in Textbox 1 on the information provided by the authors. Therefore, our classification of patient-centered digital health records might not correspond with the term used by the authors.

^b^In this column, we indicated whether authors evaluated the complete patient-centered digital health record, or only part of it.

^c^By definition, patient-centered digital health records have both passive and active features. In this column, we indicate whether patient-centered digital health records predominantly offer passive or active features. In passive features, patients receive information but do not actively add it. In terms of active features, patients perform an action and actively engage with the portal.

^d^PP: patient portal.

^e^BP: blood pressure.

^f^PHR: personal health record.

**Table 8:** Patient-centered digital health record descriptions for disease category hematological and oncological diseases (n=14).^a^

Author, year		Name		Type		What is evaluated?^b^		Passive features		Active features		Focus^c^
Cahill et al [90], 2014	MyMDAnderson		Tethered PHR^d^		PHR		View health information (including correspondence, operative reports, laboratory results, and imaging), read education material		Messaging, request prescription refills, schedule appointments		Passive	
Chiche et al [91], 2012	Sanoia		PP^e^		PP+ITP^f^ features		View health information (including allergies, vaccinations, medication, and test results), ITP-specific educational material, read emergency protocols		Messaging		Passive	
Collins et al [92], 2003	Advoy		PP		PP		View health information (treatment regimen), read educational material		Registration of symptoms and medication use, automated alerts are sent to professionals		Active	
Coquet et al [93], 2020	MyHealth portal		PP		Email use		View health information (including laboratory results)		Messaging, schedule appointments, request prescription refills, pay bills		Active	
Groen et al [94], 2017	MyAVL		PP		PP		View health information (including laboratory results, lung function, and correspondence), view appointments, read personalized information		Upload patient-reported outcomes, receive tailored physical activity advice		Active	
Hall et al [95], 2014	MyFoxChase		PP		Genetic screening		View health information (including laboratory results), view appointments, read educational material		Messaging, receive alerts if genetic screening results are available		Passive	
Hong et al [96], 2016	MyChart		PP		PP		View health information (including laboratory results, medication, allergies)		Messaging, schedule appointments, request prescription refills, use a journal		Passive	
Kidwell et al [97], 2019	MyChart		PP		PP		View health information (including laboratory results, medication, diagnoses, and allergies), view appointments, read information about sickle cell disease		Messaging		Passive	
Martinez Nicolás et al [89], 2019	Not reported		PP		PP		View health information (including laboratory results, imaging, and medication)		Messaging, teleconsulting, schedule appointments, upload glucose measurements		Active	
O’Hea et al [98], 2021	Polaris Oncology Survivorship Transition		PP		PP		View health information (including diagnoses, operative reports, and medication), view appointments, read educational material		Request a referral		Passive	
Pai et al [99], 2013	PROVIDER		Tethered PHR		PHR		View health information (including laboratory results, medication, pathology, imaging, and correspondence), read educational material		Messaging, use decision support tools, fill in questionnaires		Passive	
Tarver et al [100], 2019	OpenMRS		Tethered PHR		PHR+extra feature		View health information (including treatment history, diagnoses, and care plan), view a treatment summary, read educational material		Messaging, peer support		Passive	
Wiljer et al [101], 2010	InfoWell		Tethered PHR		PHR		View health information (including medication, laboratory results, imaging, and pathology), view appointments		Patients can organize and upload care information		Passive	
Williamson et al [102], 2017	SurvivorLink		Untethered PHR		PHR		Read educational material		Upload health documents and share these with professionals		Active	

^a^All studies are listed once in Tables 2-5 and are reported in the disease category of the condition that is most prominently investigated. We have included only the functionalities that the authors have reported in their articles. We have applied the taxonomy as presented in Textbox 1 on the information provided by the authors. Therefore, our classification of patient-centered digital health records might not correspond with the term used by the authors.

^b^In this column, we indicated whether authors evaluated the complete patient-centered digital health record, or only part of it.

^c^By definition, patient-centered digital health records have both passive and active features. In this column, we indicate whether patient-centered digital health records predominantly offer passive or active features. In passive features, patients receive information but do not actively add it. In terms of active features, patients perform an action and actively engage with the portal.

^d^PHR: personal health record.

^e^PP: patient portal.

^f^ITP: idiopathic thrombocytopenic purpura.

**Table 9:** Patient-centered digital health record descriptions for disease category other diseases (of 21 studies investigating other diseases, 20 are listed in Table 9).^a^

Author, year		Name		Type		What is evaluated?^b^		Passive features		Active features		Focus^c^
Anand et al [103], 2017	Adam’s Love		PP^d^		PP		View health information (HIV test results), receive appointment reminders		Schedule HIV test appointments, use e-counseling, receive appointment reminders		Active	
Bidmead et al [104], 2016	Patients Know Best		Tethered PHR^e^		PHR		View health information (including medication, laboratory results, and correspondence), read educational material		Communication with health care providers, upload and share health information		Active	
Crouch et al [105], 2015	My HealtheVet		PP		PP		View health information (including laboratory results and correspondence)		Messaging, request prescription refills		Passive	
Druss et al [106], 2014	MyHealthRecord		PP		PP+training		View health information (including diagnoses, measurements, laboratory results, medication, and allergies), view treatment goals		Prompts remind patients of routine preventive service		Passive	
Druss et al [77], 2020	Not reported		PP		PP+training		View health information (including medication, allergies, measurements, and laboratory results)		Formulate long-term goals, that are translated into action plans with progress tracking		Active	
Jhamb et al [107], 2015	Not reported		PP		PP		View health information (including diagnoses, allergies, immunizations, and laboratory results)		Messaging, schedule appointments, request prescription refills		Passive	
Kahn et al [108], 2010	MyHERO		PP		PP		View health information (including diagnoses, medication, laboratory results, and allergies), view appointments, read information on interpreting test results		Upload notes and self-measurements		Passive	
Keith McInnes et al [109], 2013 and Keith McInnes et al [110], 2017	My HealtheVet		PP		PP		View health information (including medication and correspondence), view appointments		Messaging, request prescription refills, receive reminders, upload notes and self-measurements, use a journal		Passive	
Kiberd et al [111], 2018	RelayHealth		PP		PP		View health information (including test results and medication)		Messaging		Active	
Lee et al [112], 2017	CoPHR		PP		PP		View health information (including diagnoses, laboratory results, medication, allergies, vital signs, and correspondence), view appointments, view treatment plan, read educational information		Manage and edit appointments and health information		Passive	
Miller et al [113], 2011	Mellen Center Care Online		Untethered PHR		PHR		Review previously entered symptoms and HRQoL^f^		Messaging, report symptoms and HRQoL and evaluate changes, preparation for appointments		Active	
Navaneethan et al [114], 2017	MyChart		PP		PP+part of users received training		View health information (including medication and laboratory results), read educational material		Messaging, schedule appointments, request prescription refills		Passive	
Plimpton [115] 2020	Not reported		PP		PP		View health information		Messaging		Passive	
Reich et al [116], 2019	MyChart		PP		PP		View health information (including laboratory results, diagnoses, medication, and vital signs)		Messaging		Passive	
Scott Nielsen et al [117], 2012	PatientSite10		PP		PP		View health information (including laboratory results, and imaging), read educational material		Messaging, schedule appointments, request prescription refills, upload self-measurements, pay bills		Active	
Son and Nahm [118], 2019	MyChart		PP		PP+training		View health information (including medication and laboratory results), read educational material		Messaging, schedule appointments, request prescription refills		Passive	
Tom et al [119], 2012	MyGroupHealth		PP		PP		View health information (including diagnoses, medication, and test results), read after-visit summaries, proxy access		Messaging, schedule appointments		Passive	
van den Heuvel et al [120], 2018	“PHR-BD”		Tethered PHR		Tethered PHR+mood chart		View health information (including diagnoses, laboratory results, medication, and correspondence), read educational material		Messaging, report symptoms in a mood chart, view personal crisis plan		Active	
van der Vaart et al [121], 2014	Not reported		PP		PP		View health information (including diagnoses, medication, and laboratory results), read educational material		Report and monitor HRQoL outcomes		Active	

^a^All studies are listed once in Tables 2-5 and are reported in the disease category of the condition that is most prominently investigated. We have included only the functionalities that the authors have reported in their articles. We have applied the taxonomy as presented in Textbox 1 on the information provided by the authors. Therefore, our classification of patient-centered digital health records might not correspond with the term used by the authors.

^b^In this column, we indicated whether authors evaluated the complete patient-centered digital health record, or only part of it.

^c^By definition, patient-centered digital health records have both passive and active features. In this column, we indicate whether patient-centered digital health records predominantly offer passive or active features. In passive features, patients receive information but do not actively add it. In terms of active features, patients perform an action and actively engage with the portal.

^d^PP: patient portal.

^e^PHR: personal health record.

^f^HRQoL: health-related quality of life.

### Outcomes

**Figure 2:**
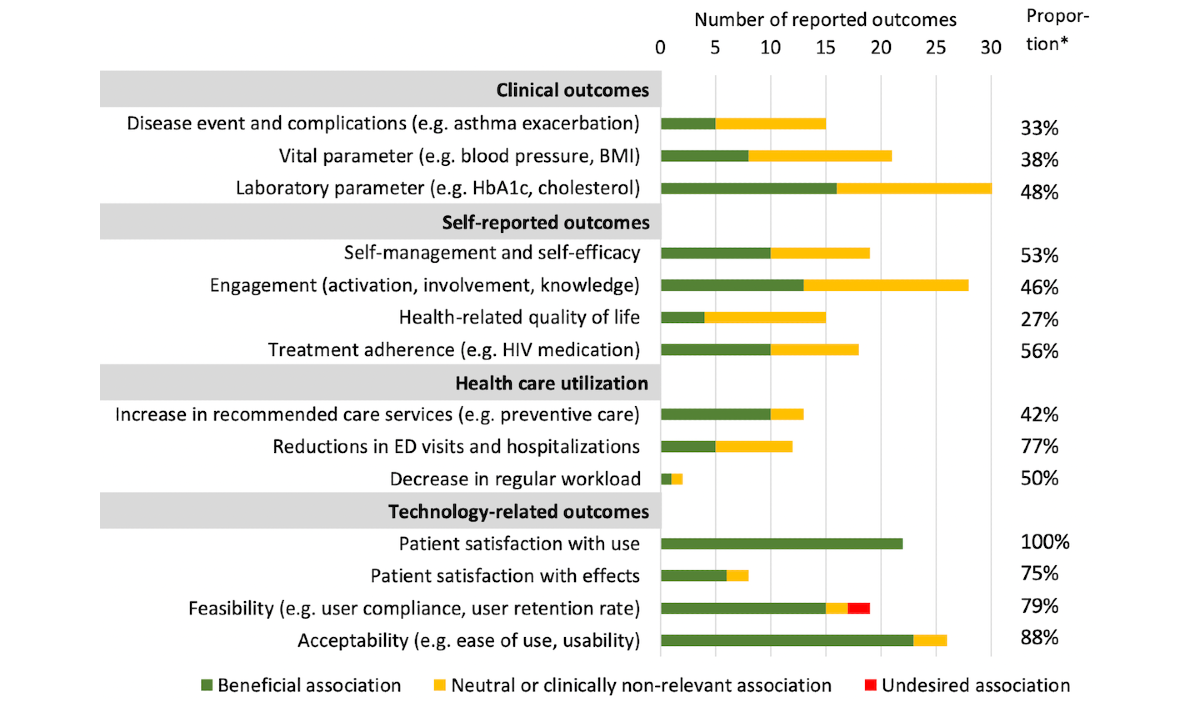
Health outcomes associated with patient-centered digital health record use. Associations refer to meaningful clinical effects or statistical significance. If studies report multiple health outcome within 1 category, each health outcome is included separately. *The proportion of health outcomes for which beneficial effects were reported. ED: emergency department.

An overview of reported associations for each health outcome is shown in Figure 2. The proportions of beneficial effects reported per health outcome are presented in Multimedia Appendices 3 and 4. For high-quality studies, proportions are presented in Multimedia Appendix 3. An overview of study conclusions and associated outcomes is presented in Tables 10-13. Studies were grouped according to disease group.

**Table 10:** Conclusions and health outcomes: all studies investigating diabetes (n=37), of which 8 (22%) are of high methodological quality.^a^

Author, year	Participants	Comparison	Main conclusion	Study design	Clinical	Patient reported	Care utilization	Technology	Quality^b^
Boogerd et al [42], 2017	Parents of children with DM^c^ type 1	PP^d^ users versus PP nonusers	Patient portal use is not associated with less parental stress. The more stress, the more parents use the portal.	QE^e^	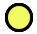	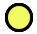	—^f^	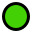	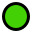
Lau et al [51], 2014	Patients with DM	Pretest PP nonuse versus posttest PP use	Patient portal use is associated with improved glycemic control.	Cohort	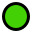	—	—	—	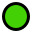
Lyles et al [52], 2016	Adults with DM type 2 using statins, registered for PP	Prescription refill use versus no refill use	Requesting prescription refills is associated with improved statin adherence.	Cohort	—	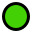	—	—	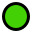
McCarrier et al [54], 2009	Adults aged <50 years with uncontrolled DM type 1	Nurse-aided PP users versus PP nonusers	Patient portal use results in improved self-efficacy, but not in improved glycemic control.	RCT^g^	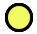	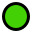	—	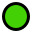	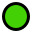
Price-Haywood and Luo [56], 2017	Adults with DM (or HT^h^)	PP users versus PP nonusers	Patient portal use is associated with more primary care visits and telephone encounters, but not with less hospitalizations or ED^i^ visits.	Cohort	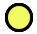	—	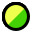	—	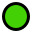
Sarkar et al [68], 2014	Adults with DM, registered for PP	Recurrent prescription refill use versus occasional refill use versus no refill use	Recurrent use of prescription refills is associated with improvements in adherence and lipid control.	Cohort	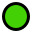	—	—	—	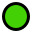
Shimada et al [71], 2016	Veterans with uncontrolled DM, registered for PP	Messaging and prescription refills users versus PP users who use neither	Messaging or requesting prescription refills is associated with improved glycemic control.	Cohort	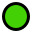	—	—	—	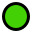
van Vugt et al [73], 2016	Patients with DM type 2, registered for PHR^j^	PHR+personal coach versus PHR use alone	PHR use does not result in improved glycemic control, self-care, distress, nor well-being, regardless of personal coaching.	RCT	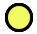	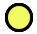	—	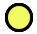	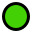
Dixon et al [47], 2016	Adults with DM type 2	Pretest PP nonusers versus posttest PP users	Patient portal use is associated with improved adherence, but not with changes in clinical outcomes nor care utilization.	QE	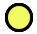	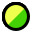	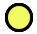	—	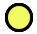
Druss et al [77], 2020	Patients with a mental disorder+DM, HT or HC^k^	PP users versus PP nonusers	Patient portal use does not result in clinically relevant improvements in perceived quality of care, patient activation nor HRQoL^l^.	RCT	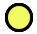	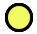	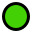	—	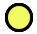
Graetz et al [49], 2020	Adults with DM with at least 1 oral drug	PP users versus PP nonusers	Patient portal use is associated with small, likely irrelevant improvements in glycemic control and medication adherence.	Cross	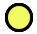	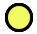	—	—	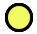
Grant et al [50], 2008	Adults with DM using medication	Tethered PP use versus untethered PP use	Using a tethered patient portal results in increased patient participation, but not improved glycemic control.	RCT	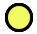	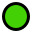	—	—	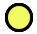
Reed et al [60], 2019	Adults with DM+HT, asthma, CAD^m^, or CHF^n^	PP users versus PP nonusers	Patient portal use is associated with more outpatient office visits, and with reduced ED visits and preventable hospitalizations.	Cross	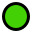	—	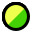	—	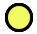
Riippa et al [62], 2014	Adults with DM, HT, or HC	PP users versus PP nonusers	Patient portal use does not result in clinically relevant improvements in patient activation, except among adults with low baseline activation.	RCT	—	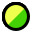	—	—	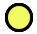
Riippa et al [63], 2015	Adults with DM, HT, or HC	PP users versus PP nonusers	Patient portal use does not result in clinically relevant improvement in patient activation nor HRQoL.	RCT	—	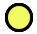	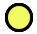	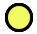	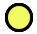
Robinsonet al [64], 2020	Veterans with uncontrolled DM type 2, registered for PP	Responders on team-initiated messages versus nonresponders	Responding on messages is associated with improved self-management and self-efficacy.	Cross	—	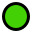	—	—	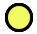
Ronda et al [65], 2014	Adults with DM	Recurrent PP users versus PP nonusers	Recurrent patient portal use is associated with better self-efficacy and knowledge.	Cross	—	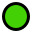	—	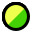	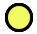
Ronda et al [66], 2015	Adults with DM, registered for PP	Persistent users versus early quitters	Recurrent users believe the patient portal increases disease knowledge, and they find it useful.	Cross	—	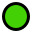	—	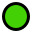	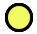
Sabo et al [67], 2021	Adults with DM type 2, registered for PP	PP users versus PP nonusers	Patient portal use has minor, clinically irrelevant effects on BMI, and no effects on glycemic control nor blood pressure.	RCT	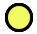	—	—	—	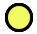
Seo et al [69], 2020	Patients with DM, registered for PHR	Continuous users versus noncontinuous users	Continuous use of a tethered PHR is associated with slightly improved glycemic control. Clinical implications are doubtful.	Cohort	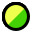	—	—	—	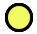
Sharit et al [70], 2018	Overweight veterans with prediabetes	Pretest PP nonuse versus posttest PP use	Using an accelerometer-connected patient portal is associated with improvements in physical activity and blood pressure.	QE	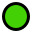	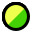	—	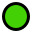	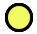
Tenforde et al [72], 2012	Adults aged <75 years with DM	PP users versus PP nonusers	Patient portal use is associated with slightly improved diabetes control, lipid profile, and blood pressure. Clinical implications are doubtful.	Cohort	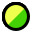	—	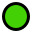	—	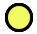
Vo et al [74], 2019	Adults aged <80 years with DM type 2, registered for PP	Previsit message use versus no previsit message use	Sending previsit prioritization messages does not result in improved glycemic control, but does result in improved perceived shared-decision-making.	RCT	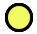	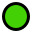	—	—	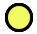
Zocchi et al [76], 2021	Patients with DM type 2, registered for PP	PP users	Among existing patient portal users with uncontrolled DM or high LDL^o^, increased use is associated with improved control.	Cohort	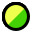	—	—	—	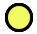
Bailey et al [41], 2019	Adults with DM, on high-risk medication	PP users	Patients are satisfied with the patient portal.	QE	—	—	—	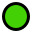	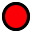
Byczkowski et al [43], 2014	Parents of children with DM (or CF^p^ or JIA^q^)	PP users	Patients consider the patient portal to be useful in managing and understand their child’s disease.	Cross	—	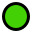	—	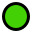	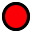
Chung et al [44], 2017	Adults with DM, registered for PP	Message users versus message nonusers	Using secure messaging is associated with better glycemic control.	Cohort	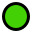	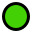	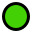	—	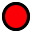
Conway et al [45], 2019	Patients with DM, registered for PP	PP users	Patients believe the tethered diabetes PHR might improve their diabetes self-care.	Cross	—	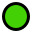	—	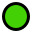	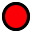
Devkota et al [46], 2016	Patients with DM type 2	PP users who read and write emails versus PP nonusers	Reading and writing emails is associated with improved glycemic control.	Cohort	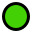	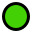	—	—	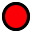
Graetz et al [48], 2018	Adults with DM	PP users versus PP nonusers	Patient portal use is associated with improved adherence to medication and preventive care utilization.	Cross	—	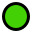	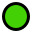	—	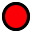
Martinez et al [53], 2021	Adults with DM type 2 using medication, registered for PP	Pretest PP nonuse versus posttest PP use	Patient portal use results in clinically not relevant improvements in patient activation and self-efficacy. This is related to the very short follow-up period of the study.	QE	—	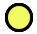	—	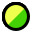	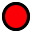
Osborn et al [55], 2013	Adults with DM type 2 using medication	PP users versus PP nonusers	Patient portal use is not associated with improved glycemic control, as compared with nonusers. However, among users, more frequent use is associated with improved glycemic control.	Cross	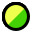	—	—	—	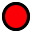
Price-Haywood et al [57], 2018	Adults with DM (or HT)	PP users versus PP nonusers	Messaging is associated with improved glycemic control.	Cohort	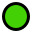	—	—	—	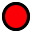
Quinn et al [58], 2018	Adults aged <65 years with DM type 2	PP+extra module users versus PP users	Messaging is associated with better glycemic control. Note: glycemic parameters were predicted and not represent measurements.	RCT	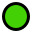				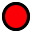
Reed et al [59], 2015	Adults with DM, HT, asthma, CAD, or CHF, registered for PP	PP users	One-third of patients report that messaging in a patient portal results in less health care visits and improved overall health.	Cross	—	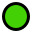	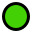	—	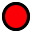
Reed et al [61], 2019	Adults with DM, asthma, HT, CAD, CHF, or CV^r^ event risk	PP users versus PP nonusers	One-third of patients report that using the patient portal improves overall health.	Cross	—	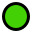	—	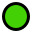	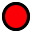
Wald et al [75], 2009	Patients with DM type 2	PHR users who created a previsit plan	Users who create a previsit care plan feel better prepared for visits.	RCT	—	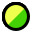	—	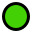	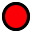

^a^Studies are listed multiple times in Tables 10-13. Per disease category, the relevant subconclusion and health outcomes are described. Associations with health outcomes are color-coded as green for beneficial, yellow for neutral or clinically nonrelevant, or red for undesired. The half green and half yellow symbol implies that one study investigated multiple outcomes in one category and reported beneficial associations for some outcomes and neutral associations for others.

^b^Quality appraisal—green: high quality; yellow: medium quality; red: low quality.

^c^DM: diabetes mellitus.

^d^PP: patient portal.

^e^QE: quasi-experimental, including pretest-posttest studies and feasibility studies.

^f^The study did not assess any health outcome in a certain category.

^g^RCT: randomized controlled trial.

^h^HT: hypertension.

^i^ED: emergency department.

^j^PHR: personal health record.

^k^HC: hypercholesteremia.

^l^HRQoL: health-related quality of life.

^m^CAD: coronary artery disease.

^n^CHF: congestive heart failure.

^o^LDL: low-density lipoprotein.

^p^CF: cystic fibrosis.

^q^JIA: juvenile idiopathic arthritis.

^r^CV: cardiovascular.

**Table 11:** Conclusions and health outcomes: studies investigating cardiopulmonary diseases (n=21), of which 6 (29%) are of high methodological quality.^a^

Author, year	Participants	Comparison	Conclusion	Study design	Clinical	Patient reported	Care utilization	Technology	Quality^b^
Ahmed et al [79], 2016	Adults with asthma using medication	PP^c^ users versus PP nonusers	Patient portal use does not result in durable improvements in HRQoL^d^ nor asthma control.	RCT^e^	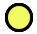	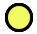	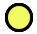	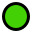	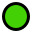
Fiks et al [81], 2015	Children aged 6-12 years with asthma	PP users versus PP nonusers	Patient portal use results in improved asthma control.	RCT	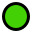	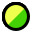	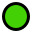	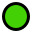	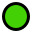
Lau et al [85], 2015	Adults with asthma	PHR^f^ users versus PHR nonusers	PHR use does not increase the use of asthma action plans, and does not affect asthma control, health care utilization nor work or school participation.	RCT	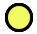	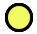	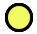	—^g^	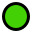
Manard et al [86], 2016	Adults with uncontrolled HT^h^	PP users versus PP nonusers	Using a patient portal linked with a blood pressure cuff is not associated with improved blood pressure control.	Cohort	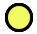	—	—	—	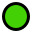
Price-Haywood and Luo [56], 2017	Adults with HT (or DM^i^)	PP users versus PP nonusers	Patient portal use is associated with more primary care visits and telephone encounters, but not hospitalizations or ED^j^ visits. Effects on blood pressure control are not clinically relevant.	Cohort	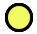	—	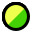	—	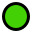
Shimada et al [71], 2016	Veterans with uncontrolled HC^k^ or HT, registered for PP	Users of both messaging and prescription refills versus nonusers	Messaging or requesting prescription refills are both associated with improved lipid control. Requesting prescription refills is associated with improved blood pressure control.	Cohort	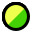	—	—	—	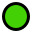
Apter et al [80], 2019	Adults with asthma using prednisone	PP use+training versus PP use+assistance via home visits	Patient portal use results in minor improvements in asthma control and HRQoL. Conducting home visits results in more improvements in these outcomes.	RCT	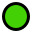	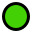	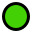	—	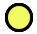
Druss et al [77], 2020	Patients with a mental disorder+DM^i^, HT^j^, or HC^k^	PP users versus PP nonusers	Patient portal use does not result in clinically relevant improvements in perceived quality of care, patient activation, nor HRQoL.	RCT	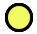	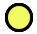	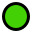	—	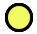
Fiks et al [82], 2016	Children aged 6-12 years with asthma	PP users versus PP nonusers	Patient portal use is associated with improved treatment adherence. Among patients with uncontrolled asthma, its use is associated with more care visits. Adoption is low.	QE^l^	—	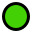	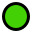	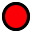	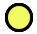
Martinez Nicolás et al [89], 2019	Patients with COPD^m^ or CHF^n^	Pretest PP nonuse versus posttest PP use	Patient portal use is associated with less hospitalizations, readmissions, and ED visits among patients with CHF and COPD.	QE	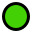	—	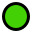	—	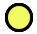
Reed et al [60], 2019	Adults with DM+HT, asthma, CAD^m^, or CHF^n^	PP users versus PP nonusers	Patient portal use is associated with more outpatient office visits, and with reduced ED visits and preventable hospitalizations.	Cross	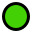	—	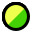	—	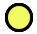
Riippa et al [62], 2014	Adults with DM, HT, or HC	PP users versus PP nonusers	Patient portal use does not result in clinically relevant improvements in patient activation, except for patients with low baseline activation.	RCT	—	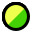	—	—	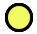
Riippa et al [63], 2015	Adults with DM, HT, or HC	Patient portal versus usual care	Patient portal use does not result in clinically relevant improvement in patient activation nor HRQoL.	RCT	—	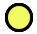	—	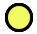	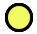
Toscos et al [87], 2020	Patients with nonvalvular AF^o^ with an oral anticoagulant drug	PP users versus PP nonusers	Using a patient portal connected to a Smart Pill Bottle does not result in improved drug adherence.	RCT	—	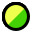	—	—	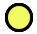
Wagner et al [88], 2012	Patients with HT	PHR users versus PHR nonusers	Using a tethered PHR does not result in clinically relevant improvements in blood pressure control, patient activation nor health care utilization. Adoption is low.	RCT	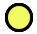	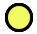	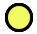	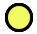	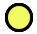
Aberger et al [78], 2014	Postrenal transplant patients with HT	PP users	Using a patient portal–linked blood pressure monitoring system is associated with improved blood pressure control.	QE	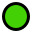	—	—	—	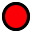
Kim et al [84], 2019	Patients with obstructive sleep apnea	PHR+activity tracker versus PHR alone versus nonusers	Using a tethered PHR results in more weight loss, regardless of its connection to an activity tracker. No sleep-related outcome improvements are seen.	RCT	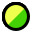	—	—	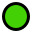	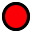
Kogut et al [83], 2014	Adults aged >49 years with cardiopulmonary disorders	PHR users versus PHR nonusers	Pharmacists reviewing patient-reported medication lists in a PHR might identify more medication-related problems.	QE	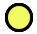	—	—	—	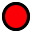
Price-Haywood et al [57], 2018	Adults with HT or DM	PP users versus PP nonusers	Messaging is not associated with improved blood pressure control.	Cohort	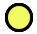	—	—	—	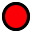
Reed et al [59], 2015	Adults with DM, HT, asthma, CAD^p^, or CHF, registered for PP	PP users	One-third of patients report that messaging in a patient portal results in less health care visits and improved overall health.	Cross-sectional	—	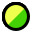	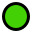	—	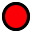
Reed et al [61], 2019	Adults with DM, asthma, HT, CAD, CHF, or CV^q^ event risk	PP users versus PP nonusers	A third of patients reports that using the patient portal improves overall health.	Cross-sectional	—	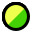	—	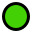	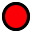

^a^Studies are listed multiple times in Tables 10-13. Per disease category, the relevant subconclusion and health outcomes are described.

^b^For color coding of quality appraisal and health outcomes, see Table 10.

^c^PP: patient portal.

^d^HRQoL: health-related quality of life.

^e^RCT: randomized controlled trial.

^f^PHR: personal health record.

^g^The study did not assess any health outcome in a certain category.

^h^HT: hypertension.

^i^DM: diabetes mellitus.

^j^ED: emergency department.

^k^HC: hypercholesteremia.

^l^QE: quasi-experimental, including pilot or feasibility studies.

^m^COPD: chronic obstructive pulmonary disease.

^n^CHF: Congestive heart failure.

^o^AF: atrial fibrillation.

^p^CAD: coronary artery disease.

^q^CV: cardiovascular.

**Table 12:** Conclusions and health outcomes: studies investigating hematological and oncological diseases (n=14), of which 2 are of high methodological quality (14%).^a^

Author, year	Participants	Comparison	Conclusion	Study design	Clinical	Patient reported	Care utilization	Technology	Quality^b^
Cahill et al [90], 2014	Adults with a brain tumor	PHR^c^ users versus PHR nonusers	Using a tethered PHR is associated with improvements in patient uncertainty.	Cross-sectional	—	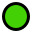	—^d^	—	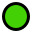
Coquet et al [93], 2020	Patients with cancer+chemotherapy, registered for PP^e^	Email users versus email nonusers	Sending emails is associated with improved 2-year survival, less missed appointments, and less hospitalizations.	Cohort	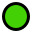	—	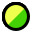	—	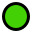
Chiche et al [91], 2012	Adults with ITP^f^	PP users versus PP nonusers	Patient portal use does not result in improved HRQoL^g^. The portal is acceptable and feasible.	RCT^h^	—	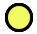	—	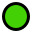	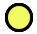
Groen et al [94], 2017	Patients with lung cancer	PP users	Patient portal use does not affect HRQoL nor patient engagement. It is feasible and acceptable.	QE^i^	—	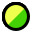	—	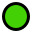	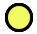
Hall et al [95], 2014	Patients with cancer resection	PP users	Disclosing results of genetic cancer screening in a patient portal might be feasible and acceptable, and is not associated with more anxiety. Yet, few abnormal results were observed.	QE	—	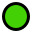	—	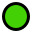	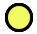
Kidwell et al [97], 2019	Patients aged 13-24 years with sickle cell disease	PP users	Patient portal use is not associated with improved medical decision-making by patients. It is acceptable and easy to use.	QE	—	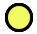	—	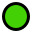	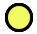
Martinez Nicolás et al [89], 2019	Patients with hematologic malignancy	Pretest PP nonuse versus posttest PP use	Patient portal use is not associated with less hospitalizations, readmissions, nor ED^j^ department visits.	QE	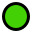	—	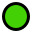	—	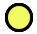
Williamson et al [102], 2017	Pediatric cancer survivors	PHR users versus PHR registrants	Patient portal use is not associated with less missed appointments.	Cohort	—	—	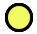	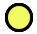	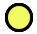
Collins et al [92], 2003	Patients with hemophilia >11 years	Users	An electronic treatment log is considered feasible and easy to use.	QE	—	—	—	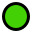	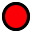
Hong et al [96], 2016	Children aged 13-17 years with cancer or a blood disorder+parents	PP users	A small cohort considers a patient portal to be feasible and useful.	Cross-sectional	—	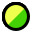	—	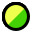	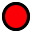
O’Hea et al [98], 2021	Women with breast cancer	PP users versus PP nonusers	Patient portal use does not result in improved HRQoL nor disease knowledge.	RCT	—	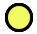	—	—	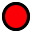
Pai et al [99], 2013	Men with prostate cancer	PHR users	Patients are satisfied with a tethered PHR and find it increases disease knowledge.	Cross-sectional	—	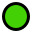	—	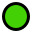	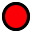
Tarver et al [100], 2019	Patients with colorectal cancer	Tethered PHR users	Patients are satisfied with an integrated care plan and find it useful.	Cohort	—	—	—	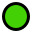	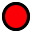
Wiljer et al [101], 2010	Patients with breast cancer	Pretest PHR nonusers versus posttest PHR users	PHR use is not associated with improved self-efficacy, nor with a clinically relevant decrease in anxiety. Satisfaction is high.	QE	—	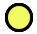	—	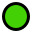	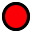

^a^Studies are listed multiple times in Tables 10-13. Per disease category, the relevant subconclusion and health outcomes are described.

^b^For color coding of quality appraisal# and health outcomes, see Table 10.

^c^PHR: personal health record.

^d^The study did not assess any health outcome in a certain category.

^e^PP: patient portal.

^f^ITP: idiopathic thrombocytopenic purpura.

^g^HRQoL: health-related quality of life.

^h^RCT: randomized controlled trial.

^i^QE: quasi-experimental, including pilot or feasibility studies.

^j^ED: emergency department.

**Table 13:** Conclusions and health outcomes: studies investigating other diseases (n=21), of which 2 (10%) are of high methodological quality.^a^

Author, year	Participants	Comparison	Conclusion	Study design	Clinical	Patient reported	Care utilization	Technology	Quality^b^
Miller et al [113], 2011	Patients with multiple sclerosis	PHR^c^ use versus PHR that only enables messaging	Using an untethered PHR results in slightly improved HRQoL^d^, but not in improved self-efficacy, disease control nor health care utilization.	RCT^e^	—	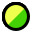	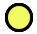	—^f^	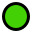
Navaneethan et al [114], 2017	Adults with chronic kidney disease	PP^g^ users+coach versus PP users versus PP nonusers	Patient portal use, regardless of added training, does not result in improved kidney function, nor altered health care utilization.	RCT	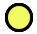	—	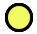	—	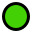
Anand et al [103], 2017	MSM^h^ and transgender women with HIV	PP users	The patient portal is feasible and acceptable.	RCT	—	—	—	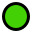	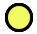
Druss et al [106], 2014	Patients with a mental disorder+chronic condition	PP users versus PP nonusers	Patient portal use results in increased use of preventive health services and medical visits, but not in improved HRQoL.	RCT	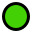	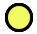	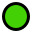	—	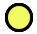
Druss et al [77], 2020	Patients with a mental disorder+DM^i^, HT^j^, or HC^k^	PP users versus PP nonusers	Patient portal use does not result in clinically relevant improvements in perceived quality of care, patient activation, nor HRQoL.	RCT	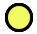	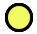	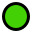	—	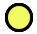
Jhamb et al [107], 2015	Adults visiting nephrology clinics	PP users versus PP nonusers	Patient portal use might be associated with improved blood pressure control, although its clinical relevance is unclear.	Cross-sectional	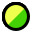	—	—	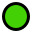	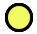
Keith McInnes et al [109], 2013	Veterans with HIV	PP users versus PP nonusers	Patient portal use is associated with improved adherence to HIV medication.	Cross-sectional	—	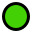	—	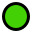	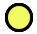
Keith McInnes et al [110], 2017	Veterans with HIV+detectable viral load, registered for PP	Messaging or prescription refill users versus nonusers	Requesting prescription refills is associated with improved HIV control, but messaging is not.	Cohort	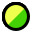	—	—	—	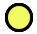
Kiberd et al [111], 2018	Adult with home dialysis	Pretest PP nonuse versus posttest PP use	Patient portal use is not associated with improvements in HRQoL nor perceived quality of care. Both were already high at baseline.	QE^l^	—	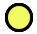	—	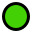	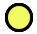
Lee et al [112], 2017	Patients with cleft lip or cleft palate surgery	PP users versus PP tailored for lip or cleft palate surgery	Using a tailored, disease-specific patient portal is associated with increased disease knowledge.	QE	—	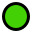	—	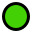	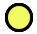
Reich et al [116], 2019	Patients with inflammatory bowel disease	PP users versus PP nonusers	Patient portal use does not result in improved HRQoL, but results in a higher vaccination rate. Patient satisfaction is high.	RCT	—	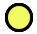	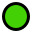	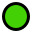	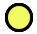
Scott Nielsen et al [117], 2012	Patients with multiple sclerosis	PP users versus PP nonusers	Messaging in a patient portal is associated with more clinic visits, but not with less ED^m^ visits nor hospitalizations.	Cross-sectional	—	—	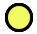	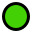	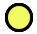
Tom et al [119], 2012	Parents of children age <6 years with 1 ore more chronic condition(s)	PP users versus PP nonusers	Patient portal use is not associated with improved access to care, nor perceived quality of care. It is considered feasible.	Cross-sectional	—	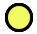	—	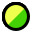	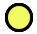
van den Heuvel et al [120], 2018	Adults with bipolar disorder	Pretest PHR nonusers versus posttest PHR users	PHR use is not associated with improved HRQoL, patient empowerment, symptom reduction, nor disease burden.	Cross-sectional	—	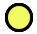	—	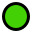	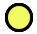
van der Vaart et al [121], 2014	Patients with rheumatoid arthritis	Pretest PP nonusers versus posttest PP users	Patient portal use is not associated with improved patient empowerment. It is considered useful and understandable.	Cross-sectional	—	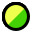	—	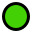	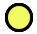
Bidmead et al [104], 2016	Patients with inflammatory bowel disease	PHR users	PHR use is not associated with improved self-management.	Cross-sectional	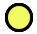	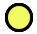	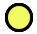	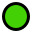	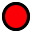
Byczkowski et al [43], 2014	Parents of children with CF^o^ or JIA^p^ (or DM)	PP users	Patients consider the patient portal to be useful in managing and understand their child’s disease.	Cross	—	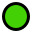	—	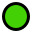	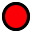
Crouch et al [105], 2015	Veterans with HIV	PP users versus PP nonusers	Patient portal use is associated with improved patient activation, disease knowledge, HIV load, but not with improved CD4-count nor treatment adherence	Cross-sectional	—	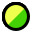	—	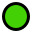	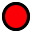
Kahn et al [108], 2010	Patients with HIV or aids	PP users	Patients are satisfied with the patient portal and consider it to be helpful in managing their problems.	QE	—	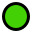	—	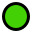	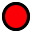
Plimpton [115], 2020	Women with HIV	Pretest PP nonuse versus posttest PP use	Patient portal use is associated with an increase in planned visits, but not with a decrease in missed visits. A trend toward improved viral load is seen.	QE	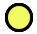	—	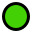	—	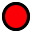
Son et al [118], 2019	Patients aged >49 years with 1 or more chronic condition(s)	PP users	Patients consider a patient portal to be helpful in increasing self-management.	Cohort	—	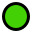	—	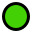	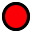

^a^Studies are listed multiple times in Tables 10-13. Per disease category, the relevant subconclusion and health outcomes are described.

^b^For color coding of quality appraisal and health outcomes, see Table 10.

^c^PHR: personal health record.

^d^HRQoL: health-related quality of life.

^e^RCT: randomized controlled trial.

^f^The study did not assess any health outcome in a certain category.

^g^PP: patient portal.

^h^MSM: men who have sex with men.

^i^DM: diabetes mellitus.

^j^HT: hypertension.

^k^HC: hypercholesteremia.

^l^QE: quasi-experimental, including pilot or feasibility studies.

^m^ED: emergency department.

^o^CF: cystic fibrosis.

^p^JIA: juvenile idiopathic arthritis.

### Clinical Outcomes

In 44 studies investigating a total of 69 clinical outcomes, a beneficial association with digital health record use was reported for 42% (29/69) of the outcomes. Hospitalizations and exacerbations were the most frequently studied disease events and complications, with beneficial effects reported in half of the studies (2/4 and 2/4, respectively). Blood pressure was the most frequently studied vital parameter, with beneficial effects reported in 36% (5/14) of the studies. HbA_1c_ and cholesterol levels were the most frequently studied laboratory parameters, with beneficial effects reported in 53% (10/19) and 57% (4/7) of the studies, respectively. No clinical outcomes were unfavorably affected by patient-centered digital health record use. In comparison with the total population, higher proportions of beneficial effects were reported for diabetes mellitus and cardiopulmonary diseases. When focusing on 14 high-quality studies, beneficial effects were observed less frequently, in only 30% (7/23) of the clinical outcomes.

Studies that assessed vital parameters generally reported few other health outcomes. However, among the studies that assessed disease events and complications, and laboratory parameters, beneficial effects were often associated with improved treatment adherence [52,68,71,81]. We hypothesize that this might be related to the removal of logistical barriers for patients in obtaining web-based prescription refills, as opposed to having to call health care providers or send them an email. Of the 6 high-quality studies that investigated treatment adherence, 2 studies assessed patient-centered digital health records that enabled patients to request prescription refills and found beneficial effects on adherence [52,68].

### Patient-Reported Outcomes

Overall, in 53 studies investigating a total of 86 patient-reported outcomes, a beneficial association with digital health record use was reported for 45% (39/86) of the outcomes. Of the 18 studies investigating 19 self-management or self-efficacy outcomes, beneficial effects were reported in 53% (9/19). Of these 9 studies, 56% (5/9) used validated questionnaires. For patient engagement outcomes, large differences in the proportions of beneficial effects were observed: from 11% (1/9) for patient activation, to 56% (5/9) for patient involvement, and 70% (7/10) for disease knowledge. However, only in measuring patient activation, validated questionnaires were principally used (8/9, 88% of studies). For HRQoL, beneficial effects were reported in 27% (4/15) of the studies, of which half used validated HRQoL questionnaires. No patient-reported outcomes were unfavorably affected by patient-centered digital health record use. In comparison to the total population, higher proportions of beneficial effects were reported for diabetes mellitus, especially for patient engagement and treatment adherence. Lowest proportions were reported for cardiopulmonary diseases, especially for patient engagement. When focusing on 10 high-quality studies, a lower proportion (7/19, 37%) of beneficial effects was observed.

We observed that improvements in patient engagement were especially facilitated by strengthening patient-professional communication; for example, through secure messaging [71,81,93]. In addition, both self-efficacy and HRQoL primarily seemed to be reinforced through the use of 2 functionalities: patient-professional communication [54,90,113] and information on disease progression [90,113].

### Health Care Utilization

For 24 studies investigating a total of 27 health care utilization outcomes, a beneficial association with digital health record use was observed for 59% (16/27) of the outcomes. The highest proportion (10/13, 77%) of beneficial effects was reported for an increased use of recommended care services. Of these 13 studies, 5 (38%) focused on recommended care services for people with uncontrolled disease, 4 (31%) on the use of preventive care services, and 4 (31%) on medical follow-up rates. In 25% (3/12) of the studies that assessed reductions in ED visits and hospitalizations, these were accompanied by an increased use of other care services, including outpatient clinic appointments and secure messaging. Compared with the total population, highest proportions of beneficial effects were reported for diabetes mellitus and hematological and oncological diseases. When focusing on 7 high-quality studies, lower proportions (3/9, 33%) of beneficial effects were observed.

### Technology-Related Outcomes

For 39 studies investigating a total of 75 technology-related outcomes, a beneficial association with digital health record use was observed for 88% (66/75) of the outcomes. All (22/22, 100%) studies reported high patient satisfaction with accessing and using digital health records. Furthermore, 75% (6/8) of the studies reported high patient satisfaction with the effects of using digital health records. High feasibility was reported by 79% (15/19) of the studies, and high acceptability by 88% (23/26) of the studies. Highest feasibility was reported for digital health records intended for people with hematological and oncological diseases. Lowest feasibility and acceptability were reported for digital health records intended for people with cardiopulmonary diseases. When focusing on 6 high-quality studies, proportions of studies that found beneficial effects were similar.

### High Disease Burden or Self-management

A subgroup of 47 studies that investigated patients with a high disease burden or high self-management was assessed. The following conditions were included: malignancies (11 studies), asthma (9 studies), HIV infection and AIDS (6 studies), hematologic conditions (5 studies), chronic kidney disease (3 studies), chronic heart failure (4 studies), mental disorders (3 studies), multiple sclerosis (2 studies), inflammatory bowel disease (2 studies), rheumatologic conditions (2 studies), insulin-dependent diabetes mellitus (2 studies), atrial fibrillation (1 study), cystic fibrosis (1 study), and posttransplant patients (1 study). In general, the digital health records assessed in this subgroup were more often tailored to specific patient populations through the addition of specialized functionalities or connected wearables.

In comparison with studies investigating patients with no high disease burden, studies investigating patients with a high disease burden reported considerably higher proportions of beneficial effects for vital parameters, patient engagement, reductions in ED visits and hospitalizations, and for all technology-related outcomes. Considerably lower proportions of beneficial effects were reported for laboratory parameters, health-related quality of life, treatment adherence, and increased use of recommended care services. For the 9 high methodological quality studies on high disease burden or self-management, the proportions of studies that found beneficial effects were roughly similar.

### Focus on Passive Versus Active Features

Of the 81 studies, 41 (51%) of the studied patient-centered digital health records focused on passive features and 40 (49%) focused on active features. In comparison with digital health records with an active focus, more beneficial effects were observed among digital health records with a passive focus for laboratory parameters (9/16, 56% vs 7/17, 41%), self-management and self-efficacy (7/11, 64% vs 3/8, 38%), patient engagement (9/15, 60% vs 4/13, 31%), and for an increased use of recommended care services (5/6, 83% vs 5/7, 71%). Compared with digital health records with a passive focus, more beneficial effects were observed among digital health records with an active focus on disease events or complications (4/10, 40% vs 1/5, 20%) and reductions in ED visits and hospitalizations (4/6, 67% vs 1/6, 17%). However, when focusing on high-quality studies, higher proportions of beneficial effects were seen for digital health records with an active focus on all clinical outcomes, patient-reported outcomes, reductions in ED visits and hospitalizations, patient satisfaction, and acceptability.

### Quality Appraisal

Of the 81 included studies, 27 (33%) studies were graded as low quality, 38 (47%) as medium quality, and 16 (20%) as high quality (Tables 10-13). Studies investigating cardiopulmonary conditions were of the highest quality, with 29% (6/21) of the studies graded as high quality. Of the 24 included RCTs, 7 (29%) were of high quality. Only 38% (9/24) of the RCTs concealed allocation to treatment groups, and 67% (16/24) used intention-to-treat analyses. Of the 57 studies with other designs, 9 (16%) were graded as high quality. Overall, 15% (12/81) of studies reported power calculations.

Among the 65 studies that were graded as medium or low quality, only 35% (23/65) used reliable or validated tools for the measurement of all their outcomes and 48% (31/65) for part of their outcomes. Of these 65 studies, 10 (15%) studies took adequate measures to limit selection bias and 17 (26%) studies used a control group or randomized participants.

When focusing on the 16 high-quality studies, 3 functionalities appeared to be the most effective: secure messaging to lower barriers in patient-professional interaction, prescription refill functions to improve medication adherence, and information provision on disease progression. In addition, in 16 high-quality studies, the proportions of beneficial effects were similar for a subgroup of studies that included older participants (mean age >55 years), which included a high number of female participants (>45%), or included a racially diverse population (<50% White participants), as compared with the total population.

## Discussion

### Principal Findings

In this systematic review, we evaluated evidence on the effects of the use of patient-centered digital health records in nonhospitalized patients with chronic health conditions on clinical and patient-reported outcomes, health care utilization, and technology-related outcomes. Beneficial effects were most frequently reported for the use of recommended care services (10/13, 77%) and for 4 patient-reported outcomes: disease knowledge (7/10, 70%), patient involvement (5/9, 56%), treatment adherence (10/18, 56%), and self-management and self-efficacy (10/19, 53%). Regarding clinical outcomes, beneficial effects were reported in 42% (29/69) of the studies. Beneficial effects were least frequently reported for disease events and complications (5/15, 33%) and health-related quality of life (4/15, 27%). For digital health records that predominantly focused on active features, higher proportions of beneficial effects on nearly all health outcomes were observed among the high-quality studies.

In this study, we observed that patient-centered digital health record use may be associated with an increased use of recommended care services. Beneficial effects on ED visits and hospitalizations were mainly observed when accompanied by an increased rate of follow-up appointments or secure messaging [60,89,93]. This might imply that reducing ED visits and hospitalizations is primarily achieved by facilitating patient-professional communication.

Beneficial effects were most often reported for patients with diabetes or cardiopulmonary disorders. We suggest 2 explanations. First, the focus of digital health records has been directed toward patients with diabetes and asthma for some time because of the sheer number of people with these conditions. This could have resulted in higher-quality patient-centered digital health records and patients who were more accustomed to their use. Second, the relative improvements in health outcomes might be smaller among patients with a condition with a high disease burden because of a higher baseline level of self-management skills and disease knowledge.

The proportions of beneficial effects varied considerably between health outcomes, which may be explained by 2 reasons. First, outcomes with a higher proportion of beneficial effects were more often the primary study outcomes than the secondary outcomes. Digital health records were more frequently tailored for these outcomes, yielding higher beneficial effects. Second, outcome assessment was generally less robust for outcomes with a higher proportion of beneficial effects, such as self-management and patient engagement, which might have resulted in more false-positive effects.

### Comparison With Earlier Evidence

Our results are more positive than those of the previous systematic reviews. This might be because of the increasing acceptance of digital health records, their improving quality, the increasing body of literature, or variations in digital health record definitions used. Two previous reviews found mixed effects on the use of portals on health outcomes and health care utilization [27] and reported positive effects on qualitatively assessed self-management in only one-third of the studies [25]. A recent systematic review that focused on portals intended for hospitalized patients found mixed results for patient engagement [26]. A systematic review that included only qualitative studies found that portal use was associated with positive effects on self-efficacy, treatment adherence, and disease knowledge [28]. In a review on eHealth interventions that aim to promote medication use, a weak association between digital health record use and health-related quality of life was observed [10]. This implies that digital health record engagement is not yet sufficient to affect patients’ overall health-related quality of life.

### Strengths and Limitations

This systematic review has several strengths. Our search strategy was comprehensive, to account for the lack of consensus in digital health record terminology. In addition, a wide variety of health outcomes were considered relevant to determine the impact of digital health record use. However, several limitations of this study must be considered. First, comparisons between studies were difficult because of the variety in evaluated functionalities. A similar diversity was observed among the reported follow-up durations, participants’ ages, study sample sizes, and outcomes. Second, because it was not possible to perform a meta-analysis owing to the heterogeneity in reported (disease-specific) outcome measurements and effects, we used the vote-counting method. Therefore, we could not report the effect estimates and indicated directions of effects [122]. Third, owing to a lack of agreement on feasibility and acceptability thresholds, much is left to the authors’ discretion. Fourth, JBI critical appraisal tools rank every item equally despite being not equally important. Finally, publication bias could have resulted in overestimation of the positive effects of patient-centered digital health records. More studies with positive results have been published. In addition, many of the included studies assessed more “mature” patient-centered digital health records, which could have overestimated the effects.

We observed that high patient satisfaction rates did not fully reflect in other health outcomes. This can be partly attributed to acquiescence bias and satisficing [123]. Moreover, satisfaction was often reduced to a narrow ease-of-use questionnaire, instead of satisfaction with the contribution to overall disease management. Finally, several studies only included recurrent users in their analyses, which could falsely increase feasibility. Moreover, these recurrent users likely experienced positive effects of using digital health records, which would have resulted in an overestimation of effects in randomized studies with no intention-to-treat analysis and in all nonrandomized studies.

The voluntary adoption of patient-centered digital health records by patients might reflect an intrinsic, preexisting motivation for self-management and care engagement bias, which may overestimate their effects. Patient-centered digital health record use could even be considered a surrogate measure for engagement [109,124,125]. Thus, it might be best to consider digital health records as vehicles for empowerment, strengthening existing self-management capabilities [126,127].

The effects of using patient-centered digital health records on health outcomes are not always direct but often depend on intermediate steps. For example, requesting prescription refills might depend on the actions performed by (slow-responding) physicians, nurses, or pharmacies. Thus, if using a digital health record would have no observable effects on health outcomes, this could also be a result of these intermediate steps or unforeseen processes and may not be attributable to the use of the patient-centered digital health record.

The proportion of beneficial effects reported in high-quality studies was lower as compared with all included studies for clinical outcomes (30% vs 42%), patient-reported outcomes (37% vs 45%), and health care utilization (33% vs 59%). Nevertheless, the proportions are clinically relevant and promising considering this newly emerging field. The observed differences might be related to 4 factors. First, the selection of motivated, well-educated, digitally minded participants might have overestimated the results in most low- and moderate-quality studies. Second, most studies did not measure ongoing user activity, and assumed that registered users became recurrent users. Third, nearly all low- and moderate-quality studies reported high dropout rates, which could overestimate acceptance rates. Finally, the lack of consensus on digital health record terminology hindered the interpretation of findings. We would advocate the use of uniform definitions, such as those presented in Textbox 1 [10,17-20].

### Future Research

Future studies should adopt additional measures to adhere to a uniform taxonomy, use log data, and limit selection bias. The exclusion of less-engaged people could further expand the digital divide between patients who are digitally proficient and those who are not, resulting in an increasingly unequal distribution of care services. We suggest that researchers include a diverse population based on age, gender, disease burden, race, education level, and health literacy [128]. Finally, further research should focus on determining which functionalities are mostly responsible for the effects on the outcomes.

### Conclusions

The use of patient-centered digital health records in chronic conditions is potentially associated with beneficial effects on several patient-reported outcomes and recommended care services in a considerable number of studied digital health records. The rates of the effects were approximately similar for different patient groups. Feasibility and acceptability were high. Our findings support further implementation of patient-centered digital health records in clinical practice. Yet, higher-quality research is needed to identify effects per disease category and per health outcome and to learn which patients might benefit from specific functionalities.

## Appendix

Multimedia Appendix 1 The search strategy used, that includes terms related to the search categories: “patient,” “intervention,” and “outcome.”.

Multimedia Appendix 2 The 4 modified Joanna Briggs Institute critical appraisal tools used in this study.

Multimedia Appendix 3 Proportion of beneficial effects reported per health outcome of all studies, presented per disease category.

Multimedia Appendix 4 The proportion of beneficial effects reported per health outcome of 16 high quality studies, presented per disease category.

## Abbreviations

ED: emergency department
JBI: Joanna Briggs Institute
PHR: personal health record
PRISMA: Preferred Reporting Items for Systematic Reviews and Meta-Analyses
PROSPERO: International Prospective Register of Systematic Reviews
RCT: randomized controlled trial
