# Supplementary material for: Patient-Centered Digital Health Records and Their Effects on Health Outcomes: Systematic Review
Source: J Med Internet Res. 2022 Dec 22;24(12):e43086. doi: 10.2196/43086 (PMC9816956; doi:10.2196/43086)
Supplement: Multimedia Appendix 1 [file jmir_v24i12e43086_app1.docx]

**Appendix 1.** The search strategy used, that includes terms related to the search categories: “patient”, “intervention”, and “outcome”.

| **Patient**  "hematologic disease" OR  "hematologic diseases"[MeSH Terms] OR  hematologic* OR  "bleeding disorder" OR  "bleeding disorders" OR  haemophilia OR  hemophilia OR  platelet* OR  "platelet disorder" OR  "platelet disorders" OR  Willebrand* OR  "sickle cell" OR  "anemia, sickle cell"[MeSH] OR  thalassemia OR  "coagulation factor" OR  "coagulation factors" OR  "coagulation disorders" OR  "coagulation disorder" OR  "factor deficiency" OR  "factor deficiencies" OR  coagulation* OR  anemia OR  thrombocytopenia* OR  thrombocytopath* OR  "bone marrow" OR  myelodysplastic OR  myeloproliferative OR  leukemia OR  lymphoma OR  "chronic health condition" OR  "chronic health conditions" OR  "chronic illness" OR  "chronic illnesses" OR  "chronic condition" OR  "chronic conditions" OR  "chronically ill" OR  "chronic disease"[MeSH] OR  "Diabetes Mellitus"[Mesh] OR  IDDM OR  T1DM OR  T1D OR  T2DM OR  T2D OR  "insulin dependent" OR  "insulin dependant" OR  Diabetes OR  Diabetic OR  Asthma* OR  "Asthma"[Mesh] OR  "Cystic fibrosis" OR | "cystic fibrosis"[MeSH] OR Cancer OR  Neoplas* OR  "neoplasms"[MeSH] OR  Malignan* OR  Tumor* OR  Tumour* OR  "Acquired Immunodeficiency Syndrome" OR  AIDS OR  HIV OR  "HIV Infections"[Mesh] OR  "congenital heart" OR  "heart condition" OR  "heart defects, congenital"[MeSH] OR  "Chronic obstructive pulmonary disease" OR  COPD OR  "Pulmonary Disease, Chronic Obstructive"[Mesh] OR  "celiac disease" OR  "Celiac Disease"[Mesh] OR  IBD OR  "inflammatory bowel disease" OR  "inflammatory bowel diseases" OR  "Inflammatory Bowel Diseases"[Mesh] OR  "ulcerative colitis" OR  "Crohn disease" OR  "crohn's disease" OR  "crohns disease" OR  Arthritis OR  "Arthritis"[Mesh] OR  Arthritic OR  "multiple sclerosis" OR  "Multiple Sclerosis"[Mesh] OR  MS OR  "Chronic kidney disease" OR  CKD OR  "Renal Insufficiency, Chronic"[Mesh] OR  "chronic renal disease" OR  "chronic kidney diseases" OR  "chronic renal diseases" OR  "chronic kidney insufficiency" OR  "chronic kidney failure" | **Intervention**  "Patient portal" OR  "patient portals" OR  "patient portals"[MeSH] OR  "Electronic health record" OR  "electronic health records"[MeSH] OR  "Health records, personal"[MeSH] OR  "patient access to records"[MeSH] OR  EHR OR  "personal health record" OR  PHR OR  "medical records"[MeSH] OR  "patient web portals" OR  "patient web portal" OR  "electronic health data" OR  "Personal health data" OR  "electronic healthcare records" OR  "electronic patient record" OR  "electronic health care record" OR  "health information systems" OR  "electronic healthcare record" OR  "electronic patient records" OR  "health information system" |
| --- | --- | --- |
|  |  | **Outcome**  Feasible OR  feasibility OR  acceptability OR  Satisfaction OR  Satisfied OR  "patient-reported outcomes" OR  "patient reported outcome measures"[MeSH] OR  self-management OR  self-efficacy OR  "patient activation" OR  "treatment adherence" OR  "health outcomes" OR  "healthcare utilization" OR  "self-management"[MeSH] OR  "Patient empowerment" OR  "power, psychological"[MeSH] OR  "patient participation" OR  "treatment adherence and compliance"[MeSH] OR  "health knowledge, attitudes, practice"[MeSH] OR  "patient perceptions" OR  "patients perceptions" OR  "patients' perceptions" OR  attitude* OR  Attitude[MeSH] |
