# Supplementary material for: Patient-Centered Digital Health Records and Their Effects on Health Outcomes: Systematic Review
Source: J Med Internet Res. 2022 Dec 22;24(12):e43086. doi: 10.2196/43086 (PMC9816956; doi:10.2196/43086)
Supplement: Multimedia Appendix 2 [file jmir_v24i12e43086_app2.docx]

**Appendix 2: The 4 modified Joanna Briggs Institute (JBI) critical appraisal tools used in this study.**

JBI Randomized Controlled Trials

|  | Yes | No | Unclear | Not applicable |
| --- | --- | --- | --- | --- |
| 1. Was true randomization used for assignment of participants to treatment groups? |  |  |  |  |
| 2. Was allocation to treatment groups concealed? |  |  |  |  |
| 3. Were treatment groups similar at the baseline? |  |  |  |  |
| 4. Were the digital health record and its functionalities well-defined? |  |  |  |  |
| 5. Was the study period well-defined? |  |  |  |  |
| 6. Was the use of the intervention well-described, including its use, number of logins and features used? |  |  |  |  |
| 7. Were possible confounders adequately addressed? |  |  |  |  |
| 8. Were outcomes assessors blind to treatment assignment? |  |  |  |  |
| 9. Were treatment groups treated identically other than the intervention of interest? |  |  |  |  |
| 10. Was follow up complete and if not, were differences between groups in terms of their follow up adequately described and analysed? |  |  |  |  |
| 11. Were participants analysed in the groups to which they were randomized? |  |  |  |  |
| 12. Were outcomes measured in the same way for treatment groups? |  |  |  |  |
| 13. Were outcomes measured in a reliable way? |  |  |  |  |
| 13.1 Clinical outcomes |  |  |  |  |
| 13.2 Self-reported outcomes |  |  |  |  |
| 13.3 Healthcare utilization |  |  |  |  |
| 13.4 Technology-related outcomes |  |  |  |  |
| 14. Were appropriate statistical analyses used? |  |  |  |  |
| 15. Were deviations from the standard RCT design accounted for in the conduct and analysis of the trial? |  |  |  |  |

Cross-sectional studies

|  | Yes | No | Unclear | Not applicable |
| --- | --- | --- | --- | --- |
| 1. Were the criteria for inclusion and exclusion in the sample clearly defined? |  |  |  |  |
| 2. Were the study subjects and the setting described in detail? |  |  |  |  |
| 3. Were the digital health record and its functionalities well-defined? |  |  |  |  |
| 4. Was the period of digital health record use well-defined? |  |  |  |  |
| 5. Was the exposure measured in a valid and reliable way, including its use, number of logins and features used? |  |  |  |  |
| 6. Were objective, standard criteria used for measurement of the condition? |  |  |  |  |
| 7. Was selection bias addressed adequately? |  |  |  |  |
| 8. Were confounding factors identified? |  |  |  |  |
| 9. Were strategies to deal with confounding factors stated? |  |  |  |  |
| 10. Were the outcomes measured in a valid and reliable way? |  |  |  |  |
| 10.1 Clinical outcomes |  |  |  |  |
| 10.2 Self-reported outcomes |  |  |  |  |
| 10.3 Healthcare utilization |  |  |  |  |
| 10.4 Technology-related outcomes |  |  |  |  |
| 11. Were appropriate statistical analyses used? |  |  |  |  |

Cohort studies

|  | Yes | No | Unclear | Not applicable |
| --- | --- | --- | --- | --- |
| 1. Were the two groups similar and recruited from the same population? |  |  |  |  |
| 2. Were the exposures (patient-centered digital health records) measured similarly to assign people to both exposed and unexposed groups? |  |  |  |  |
| 3. Were the digital health record and its functionalities well-defined? |  |  |  |  |
| 4. Was the study period well-defined? |  |  |  |  |
| 5. Was the exposure (patient-centered digital health record) measured in a valid and reliable way, including its use, number of logins and features used? |  |  |  |  |
| 6. Were confounding factors identified? |  |  |  |  |
| 7. Was selection bias addressed adequately? |  |  |  |  |
| 8. Were strategies to deal with confounding factors stated? |  |  |  |  |
| 9l Were the outcomes measured in a valid and reliable way? |  |  |  |  |
| 9.1 Clinical outcomes |  |  |  |  |
| 9.2 Self-reported outcomes |  |  |  |  |
| 9.3 Healthcare utilization |  |  |  |  |
| 9.4 Technology-related outcomes |  |  |  |  |
| 10. Was the follow up time reported and sufficient to be long enough for outcomes to occur? |  |  |  |  |
| 11. Was follow up complete, and if not, were the reasons to loss to follow up described and explored? |  |  |  |  |
| 12. Was appropriate statistical analysis used? |  |  |  |  |

Quasi-experimental studies

|  | Yes | No | Unclear | Not applicable |
| --- | --- | --- | --- | --- |
| 1. Were the participants included in any comparisons similar? |  |  |  |  |
| 2. Was selection bias addressed adequately? |  |  |  |  |
| 3. Were the digital health record and its functionalities well-defined? |  |  |  |  |
| 4. Was the study period well-defined? |  |  |  |  |
| 5. Were the participants included in any comparisons receiving similar treatment/care, other than the intervention of interest? |  |  |  |  |
| 6. Was there a control group? |  |  |  |  |
| 7. Were there multiple measurements of the outcome both pre and post the intervention/exposure? |  |  |  |  |
| 8. Was follow up complete and if not, were differences between groups in terms of their follow up adequately described and analysed? |  |  |  |  |
| 9. Were the outcomes of participants included in any comparisons measured in the same way? |  |  |  |  |
| 10. Were outcomes measured in a reliable way? |  |  |  |  |
| 10.1 Clinical outcomes |  |  |  |  |
| 10.2 Self-reported outcomes |  |  |  |  |
| 10.3 Healthcare utilization |  |  |  |  |
| 10.4 Technology-related outcomes |  |  |  |  |
| 11. Was appropriate statistical analysis used? |  |  |  |  |
