# Supplementary material for: Patient-Centered Digital Health Records and Their Effects on Health Outcomes: Systematic Review
Source: J Med Internet Res. 2022 Dec 22;24(12):e43086. doi: 10.2196/43086 (PMC9816956; doi:10.2196/43086)
Supplement: Multimedia Appendix 3 [file jmir_v24i12e43086_app3.docx]

**Multimedia Appendix 3. Proportion of beneficial effects reported per health outcome,** presented per disease category

|  | All studies  (n=81) | | | | Diabetes mellitus  (n=37) | | | | Cardiopulmonary  diseases (n=21) | | | | Hematological and  oncological diseases (n=14) | | | | Other diseases  (n=20) | | | | High disease burden (n=47) | | | |
| --- | --- | --- | --- | --- | --- | --- | --- | --- | --- | --- | --- | --- | --- | --- | --- | --- | --- | --- | --- | --- | --- | --- | --- | --- |
|  | Nr. of studies ^a^ | Nr. of outcomes ^b^ | Nr. of beneficial effects ^c^ | Proportion of beneficial  effects ^d^ | Nr. of studies ^a^ | Nr. of outcomes ^b^ | Nr. of beneficial effects ^c^ | Proportion of beneficial  effects ^d^ | Nr. of studies ^a^ | Nr. of outcomes ^b^ | Nr. of beneficial effects ^c^ | Proportion of beneficial  effects ^d^ | Nr. of studies ^a^ | Nr. of outcomes ^b^ | Nr. of beneficial effects ^c^ | Proportion of beneficial  effects ^d^ | Nr. of studies ^a^ | Nr. of outcomes ^b^ | Nr. of beneficial effects ^c^ | Proportion of beneficial  effects ^d^ | Nr. of studies ^a^ | Nr. of outcomes ^b^ | Nr. of beneficial effects ^c^ | Proportion of beneficial  effects ^d^ |
| **Clinical outcomes** | 44 | 69 | 29 | 42% | 23 | 42 | 19 | 45% | 14 | 21 | 10 | 48% | 2 | 2 | 2 | 100%* | 11 | 15 | 4 | 27% | 21 | 25 | 9 | 36% |
| 1 Disease events or complications | 14 | 15 | 5 | 33% | 2 | 2 | 1 | 50% | 7 | 7 | 4 | 57%* | 2 | 2 | 2 | 100%* | 5 | 6 | 0 | 0% | 12 | 13 | 4 | 31% |
| 2 Vital parameters | 18 | 21 | 8 | 38% | 12 | 14 | 4 | 29% | 8 | 9 | 3 | 33% | 0 | 0 | 0 | NA | 3 | 3 | 2 | 67%* | 4 | 4 | 3 | 75%* |
| 3 Laboratory parameters | 25 | 33 | 16 | 48% | 20 | 26 | 14 | 54%* | 4 | 5 | 3 | 60%* | 0 | 0 | 0 | NA | 4 | 6 | 2 | 33% | 6 | 8 | 2 | 25% |
| **Patient-reported outcomes** | 53 | 86 | 39 | 45% | 25 | 35 | 20 | 57%* | 12 | 22 | 7 | 32% | 9 | 14 | 5 | 36% | 14 | 24 | 9 | 38% | 13 | 52 | 22 | 42% |
| 4 Self-management/-efficacy | 18 | 19 | 10 | 53%* | 8 | 9 | 6 | 67%* | 2 | 2 | 1 | 50% | 3 | 3 | 1 | 33% | 5 | 5 | 2 | 40% | 12 | 12 | 6 | 50% |
| 5 Patient engagement  5a Patient activation  5b Patient involvement  5c Disease knowledge | 23  9  9  10 | 28  9  9  10 | 13  1  5  7 | 46%  11%  56%*  70%* | 9  4  2  4 | 10  4  2  4 | 5  0  2  3 | 50%  0%  100%*  100%* | 8  5  2  1 | 8  5  2  1 | 1  0  0  1 | 13%  0%  0%  100%* | 5  1  2  2 | 5  1  2  2 | 2  0  1  1 | 40%  0%  50%  50% | 6  3  3  3 | 9  3  3  3 | 5  1  2  2 | 56%*  33%*  67%*  67%* | 14  5  6  6 | 14  5  6  6 | 8  1  3  4 | 57%*  20%  50%  67%* |
| 6a Health-related quality of life  6b Reduction stress or anxiety | 15  6 | 15  6 | 4  2 | 27%  33% | 4  3 | 4  3 | 2  0 | 50%  0% | 7  0 | 7  0 | 3  0 | 43%  NA | 3  3 | 3  3 | 0  2 | 0%  67%* | 6  0 | 6  0 | 1  0 | 17%  NA | 13  4 | 13  4 | 3  2 | 23%  50%* |
| 7 Treatment adherence | 18 | 18 | 10 | 56%* | 9 | 9 | 7 | 78%* | 5 | 5 | 2 | 40% | 0 | 0 | 0 | NA | 4 | 4 | 1 | 25% | 9 | 9 | 3 | 33% |
| **Health care utilization** | 24 | 27 | 16 | 59%* | 8 | 9 | 7 | 78%* | 11 | 13 | 9 | 69%* | 3 | 4 | 3 | 75%* | 8 | 9 | 4 | 44% | 17 | 19 | 11 | 58%* |
| 8 ED visits and hospitalizations | 12 | 12 | 5 | 42% | 3 | 3 | 1 | 33% | 9 | 9 | 5 | 56%* | 2 | 2 | 2 | 100%* | 2 | 2 | 0 | 0% | 8 | 8 | 4 | 50% |
| 9 Recommended care services | 13 | 13 | 10 | 77%* | 5 | 5 | 5 | 100%* | 3 | 3 | 3 | 100%* | 2 | 2 | 1 | 50% | 6 | 6 | 4 | 67%* | 9 | 9 | 6 | 67%* |
| 10 Regular workload | 2 | 2 | 1 | 50% | 1 | 1 | 1 | 100%* | 1 | 1 | 1 | 100%* | 0 | 0 | 0 | NA | 1 | 1 | 0 | 0% | 2 | 2 | 1 | 50% |
| **Technology-related outcomes** | 39 | 75 | 66 | 88%* | 13 | 21 | 17 | 81%* | 7 | 10 | 7 | 70% | 10 | 23 | 21 | 91%* | 14 | 24 | 23 | 96%* | 31 | 59 | 55 | 93%* |
| 11a Patient satisfaction with use  11b Patient satisfaction with effects | 22  8 | 22  8 | 22  6 | 100%*  75%* | 7  1 | 7  1 | 7  0 | 100%*  0% | 2  0 | 2  0 | 2  0 | 100%*  NA | 7  2 | 7  2 | 7  2 | 100%*  100%* | 7  5 | 7  5 | 7  4 | 100%*  80%* | 17  7 | 17  7 | 17  6 | 100%*  86%* |
| 12 Feasibility | 19 | 19 | 15 | 79%* | 5 | 5 | 3 | 60%* | 4 | 4 | 2 | 50% | 4 | 4 | 4 | 100%* | 6 | 6 | 6 | 100%* | 14 | 14 | 13 | 93%* |
| 13 Acceptability | 26 | 26 | 23 | 88%* | 8 | 8 | 7 | 88%* | 4 | 4 | 3 | 75% | 10 | 10 | 8 | 80%* | 6 | 6 | 6 | 100%* | 21 | 21 | 19 | 90%* |

Numbers and percentages in the disease-specific columns do not add up to the numbers and percentages in the ‘all diseases’ columns, since one study can focus on multiple diseases. Similarly, numbers and percentages in the individual health outcomes rows (e.g. disease events/complications and vital parameters) do not add up to the numbers and percentages presented in the outcome category rows (e.g. clinical outcomes), since one study can investigate multiple health outcomes.

Health outcomes for which beneficial effects were reported for >50% are marked with an *. For subgroups in which a higher proportion of beneficial effects was observed, compared to all studies, percentages are shown in green. If a lower proportion was observation, percentages are shown in red.

a The number of studies that investigated a respective health outcome.

b The total number of outcomes that were investigated within this health outcome category.

c The number of studies that reported a beneficial effect for this respective health outcome.

d The proportion of health outcomes for which a beneficial effect was found, as a fraction of the total number of health outcomes investigated within an outcome category.

Abbreviations: compl,, complications; ED, emergency department; HRQoL, health-related quality of life; NA, not applicable
